# Tissue-Autonomous Function of *Drosophila Seipin* in Preventing Ectopic Lipid Droplet Formation

**DOI:** 10.1371/journal.pgen.1001364

**Published:** 2011-04-14

**Authors:** Yuan Tian, Junfeng Bi, Guanghou Shui, Zhonghua Liu, Yanhui Xiang, Yuan Liu, Markus R. Wenk, Hongyuan Yang, Xun Huang

**Affiliations:** 1 Key Laboratory of Molecular and Developmental Biology, Institute of Genetics and Developmental Biology, Chinese Academy of Sciences, Beijing, China; 2 Graduate School of Chinese Academy of Sciences, Beijing, China; 3 Life Science Institute, National University of Singapore, Singapore, Singapore; 4 Department of Biochemistry, Yong Loo Lin School of Medicine, National University of Singapore, Singapore, Singapore; 5 Department of Biological Sciences, Yong Loo Lin School of Medicine, National University of Singapore, Singapore, Singapore; 6 School of Biotechnology and Biomolecular Sciences, University of New South Wales, Sydney, Australia; University of California San Francisco, United States of America

## Abstract

Obesity is characterized by accumulation of excess body fat, while lipodystrophy is characterized by loss or absence of body fat. Despite their opposite phenotypes, these two conditions both cause ectopic lipid storage in non-adipose tissues, leading to lipotoxicity, which has health-threatening consequences. The exact mechanisms underlying ectopic lipid storage remain elusive. Here we report the analysis of a *Drosophila* model of the most severe form of human lipodystrophy, Berardinelli-Seip Congenital Lipodystrophy 2, which is caused by mutations in the *BSCL2/Seipin* gene. In addition to reduced lipid storage in the fat body, *dSeipin* mutant flies accumulate ectopic lipid droplets in the salivary gland, a non-adipose tissue. This phenotype was suppressed by expressing dSeipin specifically within the salivary gland. *dSeipin* mutants display synergistic genetic interactions with lipogenic genes in the formation of ectopic lipid droplets. Our data suggest that dSeipin may participate in phosphatidic acid metabolism and subsequently down-regulate lipogenesis to prevent ectopic lipid droplet formation. In summary, we have demonstrated a tissue-autonomous role of dSeipin in ectopic lipid storage in lipodystrophy.

## Author Summary

Obesity and lipodystrophy are medical conditions characterized by excess body fat or too little body fat, respectively. Interestingly, a common feature of both conditions is ectopic accumulation of lipids (fat) in cells where fat is not normally stored. This can cause tissue damage with health-threatening consequences. We are trying to understand how these two very different diseases lead to lipid storage in non-fat tissues. In this study, we used fruit flies (*Drosophila melanogaster*) with a mutation in the *dSeipin* gene as a lipodystrophy model to explore the mechanism of ectopic lipid storage. In *dSeipin* mutant flies, we found numerous lipid droplets in the salivary gland, a non-fat storage tissue, and reduced lipid storage in the fat body, an adipose tissue. Furthermore, we proved that dSeipin functions within salivary gland cells to prevent the formation of ectopic lipid droplets. We also found that *dSeipin* genetically interacts with other fat synthesis and metabolism genes in the formation of ectopic lipid droplets. The fruit fly *dSeipin* mutant provides an excellent model system for dissecting the mechanisms that regulate the storage of excess lipids.

## Introduction

Lipids are major membrane components as well as the main source of cellular energy. Cells have developed precise homeostatic mechanisms to tightly regulate lipid uptake, synthesis, storage, and usage [1], [2]. Abnormalities in lipid metabolism often lead to disease states: excess body fat can lead to obesity while loss or absence of body fat results in lipodystrophy [3]. In vertebrates, white adipose tissue is the main lipid storage organ; however, ectopic lipid storage in non-adipose tissues such as muscle, pancreas, and liver, is often observed in disease states such as obesity and lipodystrophy. Lipotoxicity as a result of ectopic lipid storage in these diseases is thought to be a major cause of severe pathological conditions including insulin resistance, pancreatic β-cell failure, and hepatic steatosis [4]–[6].

Ectopic lipid storage could be due to cell-extrinsic effects or cell-intrinsic effects. Extrinsically, overflow of excess lipids that can no longer be stored in adipose tissues leads to lipid droplet formation in non-adipose tissues [7], [8]. In one study, surgical implantation of normal adipose tissue back into lipoatrophic mice reversed ectopic lipid accumulation in the liver, suggesting a tissue-non-autonomous mechanism [9]. On the other hand, defects within non-adipose tissues have also been postulated to contribute to ectopic lipid storage intrinsically, suggesting a positive role of non-adipose tissue in lipid storage. For example, removal of the transcription factor PPAR-δ specifically from cardiomyocytes results in decreased fatty acid oxidation and severe lipid storage in the heart [10]. Currently, the contribution of extrinsic and intrinsic mechanisms to ectopic lipid storage in non-adipose tissues in various diseases conditions remains to be determined.

Berardinelli-Seip Congenital Lipodystrophy (BSCL), the most severe form of lipodystrophy in humans, is caused by mutations in either *BSCL1* or *BSCL2*. *BSCL1* encodes acylglycerol phosphate acyltransferase 2 (AGPAT2), which is involved in triacylglycerol (TAG) biosynthesis, while *BSCL2* encodes Seipin [11], [12]. Although *BSCL1* and *BSCL2* patients exhibit similar disease pathology, the causal link between Seipin and AGPAT2 is unclear. *BSCL2* patients are born with nearly no adipose tissue and have ectopic lipid storage in muscle and liver, implying that human Seipin (hSeipin) may be required for adipocyte survival or differentiation [13], [14].

Within cells, lipids are stored in specialized organelles called lipid droplets. The surface of the lipid droplet is a monolayer of polar lipids in which are embedded coat proteins that may be important for lipid homeostasis. The core of the lipid droplet contains neutral lipids, predominantly TAG and sterol esters [15]–[17]. Recent studies in both yeast and human cells suggested that Seipin may regulate the morphogenesis of lipid droplets. Yeast *Seipin* mutant cells contain irregular clustered lipid droplets and sometimes giant lipid droplets, while human *BSCL2/Seipin* mutant fibroblasts were found to contain numerous small lipid droplets [18], [19]. Nevertheless, the exact role of Seipin in human and yeast remains obscure. In addition, there is no plausible explanation for the cause of the ectopic lipid storage within non-adipose tissues in human *Seipin* patients. The lack of adipose and non-adipose tissues in yeast makes it impossible to uncover the mechanisms of ectopic lipid storage using yeast mutants. Therefore, it is important to utilize suitable model organisms that have both adipose and non-adipose tissues to study Seipin function and gain insights into the underlying mechanisms of ectopic lipid storage and lipid droplet formation.

As a multi-cellular model organism, *Drosophila* and its cell lines have been widely used to study conserved mechanisms of lipid metabolism [20]–[25]. Here we report that *Drosophila Seipin* (*dSeipin*) mutants have reduced lipid storage in the fat body, an adipose tissue, while exhibiting ectopic lipid droplets in the salivary gland, a non-adipose tissue. We also found that *dSeipin* functions tissue-autonomously in both tissues. Further genetic and lipidomic analyses revealed that in the salivary gland *dSeipin* genetically interacts with lipogenic genes likely at the level of phosphatidic acid (PA). Our studies uncover an unexpected tissue-autonomous mechanism in ectopic lipid storage and provide an attractive model system with which to dissect lipid metabolism in vivo.

## Results

### *Drosophila Seipin* mutants have reduced lipid storage in the fat body

**Figure 1:**
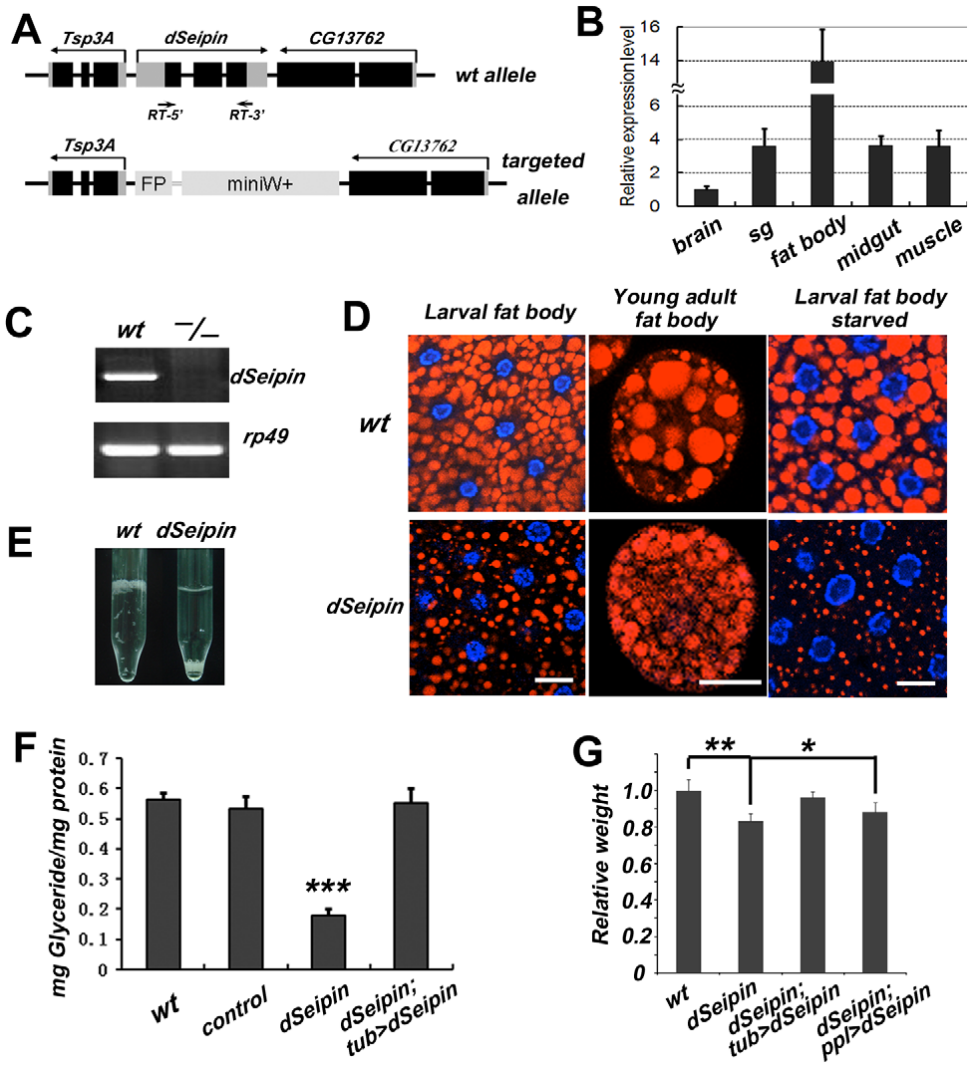
*dSeipin* mutants exhibit reduced lipid storage in the fat body. (A) Schematic of the genomic structures of wild type *dSeipin* and the null mutant. In the null mutant, the *dSeipin* locus is replaced by *GFP (FP)* and *miniWhite (miniW+)* sequences. Except for the *FP* and *miniW+* regions, black boxes represent coding regions and grey boxes represent un-translated region. (B) Expression levels of *dSeipin* in different larval tissues by qRT-PCR. The error bars represent standard deviation. (C) RT-PCR analysis of wild-type and *dSeipin* knockout larvae. Primer positions (RT-5′ and 3′) are labeled in (A). (D) Lipid droplets labeled by Nile red (red) in larval fat bodies and young adult fat cells from wild type and *dSeipin* mutants. Nuclei were stained with DAPI (blue). *dSeipin* mutants exhibit lipid storage defects with small lipid droplets. Under starved conditions the lipid droplets in *dSeipin* mutant larvae are even smaller. Scale bars: 20 µm. (E) Larval fat bodies from wild type float on top of 2% sucrose solution, while fat bodies from *dSeipin* mutants sink to the bottom. (F) Glyceride levels in adult males of wild type, control, *dSeipin* mutants and transgene-rescued *dSeipin* mutants. *dSeipin* mutants have significantly lower levels of glyceride. The error bars represent standard deviation. ***: P<0.0001. (G) Average weights (adult male) from wild type, *dSeipin* mutants and transgene-rescued *dSeipin* mutants. *dSeipin* mutants are slightly lower in weight. The error bars represent standard deviation. **: P<0.001; *: P<0.05.

A single *Drosophila* homolog of human Seipin, CG9904, was identified through Blast sequence comparisons and named as dSeipin (Figure 1A and Figure S1). The middle portion of dSeipin (aa 45–277), including two potential transmembrane domains, shares 40% identity and 63% similarity to hSeipin. To study the function of dSeipin, we examined the expression profile of *dSeipin* mRNA using quantitative-RT-PCR (qRT-PCR). We found that *dSeipin* was widely expressed in many tissues with the highest expression in the fat body and moderate expression in the salivary gland, midgut and muscle (Figure 1B). We also examined the expression of *dSeipin* mRNA through in situ hybridization. At late embryonic stages, *dSeipin* mRNA is highly expressed in the hindgut. At larval stages, *dSeipin* mRNA is expressed in the fat body, anterior midgut and salivary gland (Figure S1).

As an entry point to explore the function of dSeipin in vivo, we analyzed the subcellular localization of YFP-tagged dSeipin using the *UAS-Gal4* system, where the target gene downstream of the *UAS* element is activated by the transcriptional activator Gal4. We confirmed that the YFP-tagged dSeipin protein is functional (data not shown). We then used a fat body- and salivary gland-specific *Gal4* driver, *ppl-Gal4* [26], to express dSeipin-YFP in the fat body. dSeipin-YFP is colocalized with the ER marker PDI-GFP (Figure S1). In addition, dSeipin-YFP forms some puncta which might represent specific subdomains of the ER (Figure S1). The similar subcellular localization patterns of dSeipin to its human and yeast counterparts [18] imply that dSeipin may perform conserved roles in the regulation of lipid droplet formation and lipid storage.

We next examined the role of *dSeipin* through mutant phenotypic analysis. We generated a deletion allele of *dSeipin* using an ends-out gene targeting approach. This allele lacks the whole genomic region of *dSeipin* and is a null allele as confirmed by RT-PCR (Figure 1A and 1C). *dSeipin* mutants are viable and fertile with no noticeable behavior defects. We used the neutral lipid stain Nile red to examine the morphology of the fat body, which is the adipose tissue of insects and also has liver-like activity due to its detoxification function. The larval fat bodies of *dSeipin* mutants have significantly reduced lipid storage, in contrast to wild-type fat bodies which have many large lipid droplets (Figure 1D). Similarly, in fat cells from young adults, *dSeipin* mutants have smaller lipid droplets compared to wild type. The small lipid droplet phenotype is more severe under starvation conditions (Figure 1D). This lipid droplet phenotype is similar to that of *hSeipin* mutant fibroblasts, which also contain small lipid droplets [18]. Further genetic rescue experiments supported the idea that the lipid storage phenotype is indeed due to the deletion of *dSeipin* (see below). These results indicate that dSeipin is required for proper lipid storage in the fat body. Except for the aberrant lipid droplets, the overall morphology of the fat body in the mutants appears normal.

Interestingly, the fat bodies from *dSeipin* mutants sink to the bottom of 2% sucrose solution while control fat bodies float on top (Figure 1E). This phenomenon is likely due to reduced lipid storage in the mutant fat bodies. We examined the total glyceride content and found that the glyceride level in *dSeipin* mutants is greatly reduced compared to control animals (Figure 1F). This phenotype can be rescued by ubiquitous expression of a *UAS-dSeipin* transgene (Figure 1F). *dSeipin* mutants are viable and fertile, but their average weight is lower than control flies (Figure 1G). The weight reduction phenotype can be fully rescued by expressing *UAS-dSeipin* ubiquitously with *tub-Gal4* and partially rescued by expressing *dSeipin* in the fat body and salivary gland with *ppl-Gal4* (Figure 1G).

### *dSeipin* mutants likely have reduced lipogenesis in the fat body

**Figure 2:**
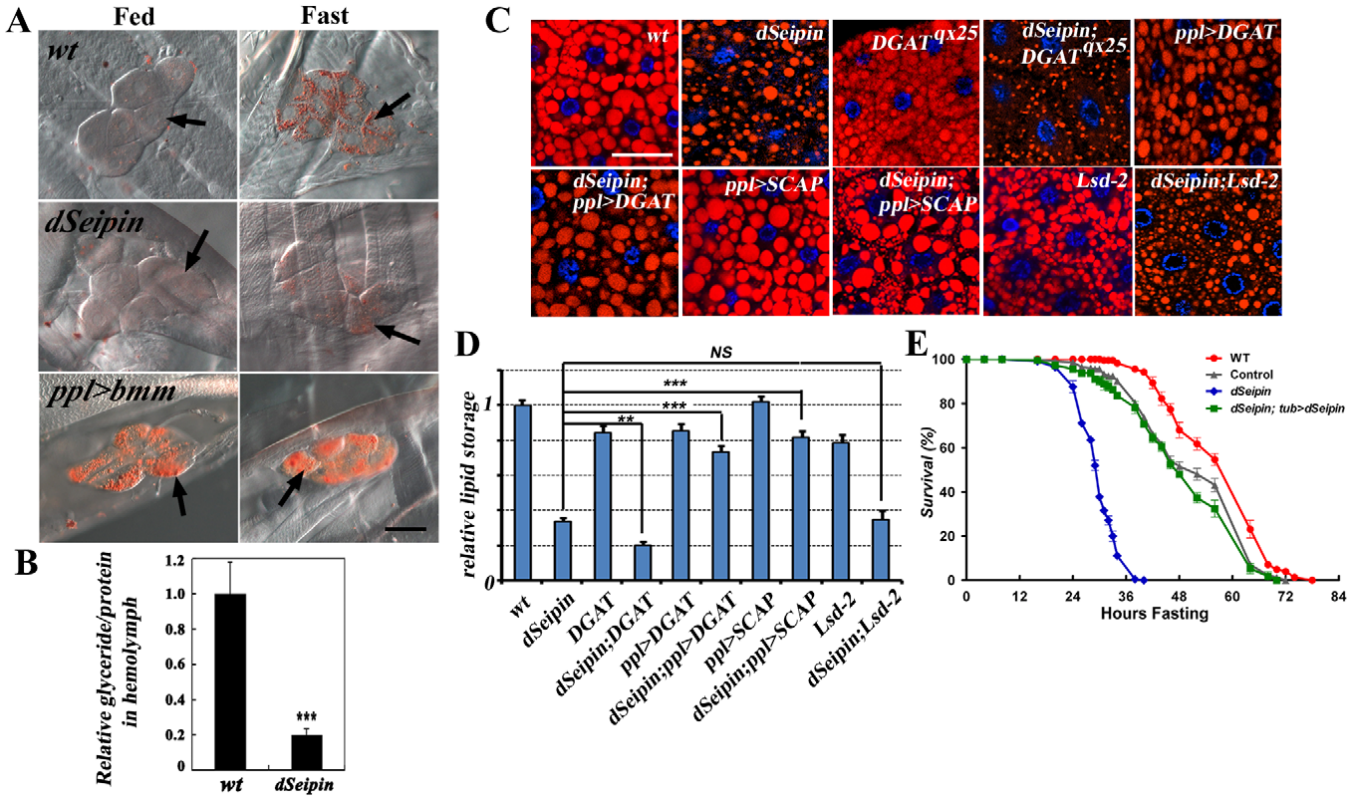
*dSeipin* mutants may have reduced lipogenesis in the fat body. (A) Oil Red O staining of oenocytes (arrows). Increasing lipolysis by overexpressing *bmm* (*ppl>bmm*) causes a strong Oil Red O signal in fed animals. *dSeipin* mutants have the same level of Oil Red O staining as wild type under fed conditions. Scale bar: 50 µm. (B) Glyceride levels in circulating hemolymph are greatly reduced in *dSeipin* mutants. ***: P<0.0001. (C) Genetic interaction of *dSeipin* with other lipid metabolism regulators. The *ppl-Gal4* driver was used to overexpress DGAT or SCAP. The genotypes are as follows: *wt*, *dSeipin*, *DGAT^qx25^*, *dSeipin;DGAT^qx25^*, *ppl>DGAT*, *dSeipin;ppl>DGAT*, *ppl>SCAP*, *dSeipin;ppl>SCAP*, *Lsd-2^KG00149^* and *dSeipin;Lsd-2^KG00149^*. *ppl>DGAT* is short for *ppl-Gal4>UAS-DGAT*, where DGAT is overexpressed using the *ppl-Gal4* driver. Scale bar: 50 µm. (D) Quantification of the genetic interactions in (C). The lipid storage in wild type was set as 100%. The error bars represent standard deviation. NS: Non-significant; ***: P<0.0001; **: P<0.001. (E) Adult starvation test. The x-axis shows the hours of starvation and the y-axis shows the survival rate (%). *dSeipin* mutants are very sensitive to starvation compared to wild type and control.

What is the cause of reduced lipid storage in *dSeipin* mutants? Both increased lipolysis and reduced lipogenesis could potentially lead to the same reduced lipid storage phenotype. Results from the following experiments rule out the former possibility. We firstly investigated the levels of circulating lipids, which are elevated if lipolysis is increased. It has been previously reported that oenocytes (larval secretory cells) can be used to monitor the levels of circulating lipids [27]. Under fed conditions in wild type, oenocytes are weakly stained with the neutral lipid dye Oil Red O because of low circulating lipids and low levels of TAG biosynthesis in oenocytes. However, under starved conditions, lipolysis is stimulated in the fat body, resulting in high levels of circulating lipids and strong Oil Red O staining of oenocytes. Moreover, under fed conditions, increasing lipolysis by overexpression of BMM, the *Drosophila* homolog of mammalian adipocyte triglyceride lipase (ATGL) [28], leads to strong Oil Red O staining in oenocytes [27] (Figure 2A). We found that under fed conditions, there is no difference between Oil Red O staining of oenocytes in wild type and *dSeipin* mutants (Figure 2A). Furthermore, in starved *dSeipin* mutants, although the Oil Red O staining signal is higher than in fed mutants, it is still much lower than that of wild type, reflecting the fact that less lipid is stored in the fat body of *dSeipin* mutants (Figure 2A). To rule out the possibility that loss of *dSeipin* in oenocytes prevents them synthesizing TAG, we specifically expressed *dSeipin* in oenocytes with *Cyp4g1-Gal4* in *dSeipin* mutants. We found that under starved conditions the Oil Red O staining signal is much lower than that of wild type (Figure S3). These results indicate that circulating lipids in *dSeipin* mutants are not elevated under normal fed conditions. Moreover, we also measured the circulating glyceride levels and found it is significantly lower in *dSeipin* mutants than wild type, supporting the idea that lipolysis is not increased in *dSeipin* mutants (Figure 2B). LSD-2 is one of the two *Drosophila* lipid droplet surface PAT domain-containing proteins. *Lsd-2* mutants may have slightly increased lipolysis [29], but *dSeipin;Lsd-2* double mutants exhibit a similar phenotype to *dSeipin* single mutants, suggesting that *dSeipin* likely does not genetically interact with *Lsd-2* (Figure 2C). Taken together, these results indicate that the reduced lipid storage in *dSeipin* mutant fat bodies is unlikely to be due to increased lipolysis.

To test whether the reduced lipid storage phenotype is due to reduced lipogenesis in the mutants, we examined the genetic interaction of *dSeipin* with several known lipid storage regulators in *Drosophila*. *Drosophila midway (mdy)* encodes a diglyceride acyltransferase (DGAT), which is involved in the last step of TAG biosynthesis [30]. In a partial loss-of-function *DGAT/mdy^qx25^* mutant alone, there is little effect on fat body lipid storage (Figure 2C and 2D). However, double mutants of *dSeipin* and *DGAT* have greatly reduced lipid storage compared to either single mutant (Figure 2C and 2D). Moreover, overexpression of DGAT or SCAP (SREBP cleavage activating protein), key positive regulators of lipogenesis [24], suppresses the reduced lipid storage phenotype in *dSeipin* mutants (Figure 2C and 2D). Taken together, these results suggest that *dSeipin* mutants likely have reduced lipogenesis in the fat body.

Since stored lipid is an important energy supply during starvation stress, reduced lipid storage might be deadly during nutrient deprivation. We therefore tested whether *dSeipin* mutants are sensitive to starvation. Under starved conditions, more than half of wild type or controls survive for at least 48 hours, while 100% of *dSeipin* mutants die within 42 hours, indicating that *dSeipin* mutants are hypersensitive to starvation (Figure 2E). The reduced lipid storage and the increased sensitivity to starvation in *dSeipin* mutants raise the possibility that *dSeipin* mutants are always in a starved state under normal fed conditions. In wild-type flies, starvation can trigger autophagy in the fat body, so if *dSeipin* mutants are always in a starved state, the autophagy program should be active under normal culturing conditions. We used the conventional lysosomal dye lysotracker to detect autophagic cells; however, we found no difference in lysotracker staining between wild type and *dSeipin* mutants. Fat bodies from both genotypes were positively stained under starved conditions and negatively stained in normal conditions, suggesting that under normal feeding conditions *dSeipin* mutants are not limited in nutrition (Figure S2).

### Ectopic lipid droplets in *dSeipin* mutants

Besides reduced lipid storage in adipose tissue, another prominent phenotype of lipodystrophy is ectopic lipid storage in non-adipose tissues, such as muscle and liver. Therefore, we also performed lipid staining in other tissues from *dSeipin* mutants, including wing disc, gut, brain, muscle, epidermis and salivary gland. Among these tissues, gut and wing disc can store lipids under normal or fasting conditions [27]. Similar to the fat body, *dSeipin* mutants have reduced lipid storage in the wing disc (Figure S2). We did not find excess lipid storage in the brain, muscle and epidermis of *dSeipin* mutants (Figure S2).

**Figure 3:**
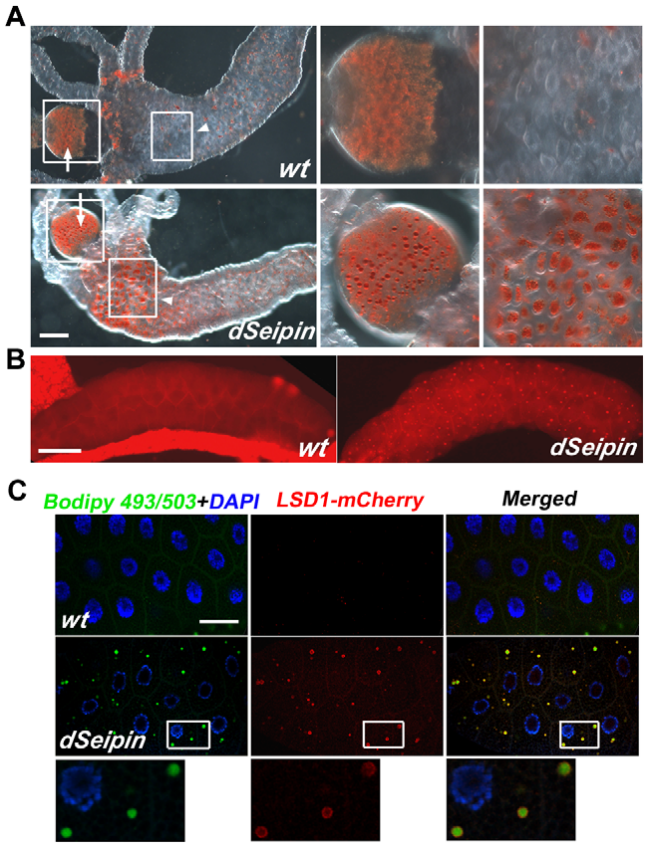
Ectopic lipid droplet formation in *dSeipin* mutants. (A) Oil Red O staining in guts of wild type and *dSeipin* mutants. In wild type the proventriculus (arrow) has numerous small droplets and the anterior midgut (arrowhead) has only a few Oil Red O-positive patches. In *dSeipin* mutants, the droplets in the proventriculus (arrow) are larger than wild type and in the anterior midgut (arrowhead) there are more patches of Oil Red O-positive staining. The boxed regions of the proventriculus and the midgut are enlarged in the adjacent panels. Scale bar: 100 µm. (B) Nile red staining in salivary glands. In wild-type salivary glands, essentially no punctate Nile red staining was found. In *dSeipin* mutants, many Nile red-positive puncta are present, indicating ectopic lipid storage. Scale bar: 100 µm. (C) Ectopic lipid puncta marked by Bodipy 493/503 (green) in *dSeipin* mutant salivary glands are confirmed as lipid droplets by co-labeling with the lipid droplet surface marker LSD-1-mCherry (red). Nuclei were stained with DAPI (blue). Scale bar: 50 µm. The lower panels show enlarged views from a *dSeipin* mutant. Expression of LSD-1-mCherry in a wild-type background was used as a control.

Interestingly, we found that the proventriculus had large lipid droplets in *dSeipin* mutants compared to small lipid droplets in wild type (Figure 3A). In the anterior midgut region, *dSeipin* mutants had much more stored lipid than wild type (Figure 3A). Moreover, we found that *dSeipin* mutants exhibit ectopic lipid droplets in the salivary gland, which normally lacks any visible lipid droplets and has never been found to serve as a lipid storage organ under any conditions. In wild-type salivary glands, Nile red staining is diffused in the cytoplasm, while in mutants many small Nile red-positive puncta were found (Figure 3B). The ectopic puncta in mutant salivary glands are bona fide lipid droplets because the lipid droplet surface marker LSD-1-mCherry forms typical ring-like structures surrounding them (Figure 3C). Together, our data show firstly that the *Drosophila dSeipin* mutation results in reduced lipogenesis and lipid storage in adipose tissue, and secondly that it causes ectopic lipid droplets in some non-adipose tissues.

### *dSeipin* is required tissue-autonomously for ectopic lipid droplet formation in salivary gland

Since the ectopic lipid droplet phenotype has not been reported before in *Drosophila*, we decided to take a genetic approach to tackle the underlying mechanisms using the salivary gland and the gut as models. First, we asked whether the ectopic lipid storage in *dSeipin* mutants is caused by intrinsic tissue-autonomous mechanisms or extrinsic tissue-non-autonomous mechanisms.

**Figure 4:**
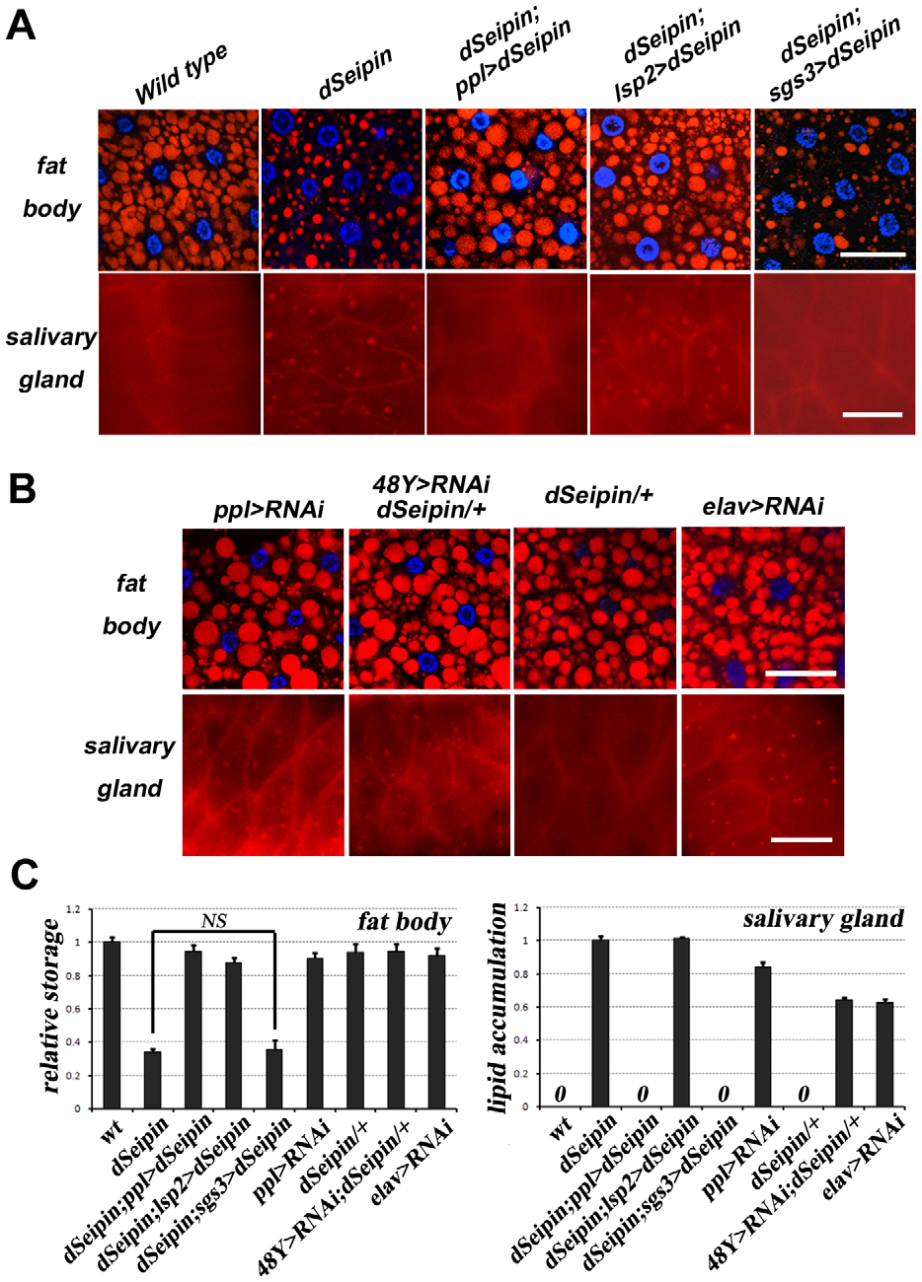
*dSeipin* functions tissue-autonomously in both salivary gland and fat body. (A) Tissue-specific rescue reveals tissue-autonomous function of *dSeipin*. Red: Nile red staining; blue: DAPI staining. The genotypes are indicated. *ppl-Gal4* drives expression in both fat body and salivary gland. *lsp2-Gal4* drives expression only in fat body, while *sgs3-Gal4* drives expression only in salivary gland. *lsp2>dSeipin* is short for *lsp2-Gal4>UAS-dSeipin*, where dSeipin is overexpressed using the *lsp-2-Gal4* driver. Note that *dSeipin;lsp2>dSeipin* animals have ectopic lipid storage in the salivary gland but exhibit normal lipid storage in the fat body and *dSeipin;sgs3>dSeipin* animals have lipid storage defects in the fat body but no lipid storage in the salivary gland. Scale bar: 50 µm. (B) Tissue-specific RNAi confirms the tissue-autonomous function of *dSeipin*. Red: Nile red staining; blue: DAPI staining. *UAS-dSeipin RNAi* driven by salivary gland-specific *48Y-Gal4* or *elav-Gal4* results in ectopic lipid storage in the salivary gland without affecting lipid storage in the fat body. Scale bar: 50 µm. (C) Quantification of (A) and (B). The error bars represent standard deviation. NS: non-significant in Student's T-test.

To test in which tissue *dSeipin* function is required, we used the *UAS-Gal4* system to express wild-type *dSeipin* in a tissue-specific manner in an otherwise *dSeipin* mutant background. Tissue-specific expression of *dSeipin* was also verified by qRT-PCR (Figure S3). *UAS-dSeipin* driven by *ppl-Gal4* (which is highly active in salivary gland and moderately active in fat body) rescued both lipid storage defects in the fat body and ectopic lipid storage in the salivary gland (Figure 4A, 4C and Table S1). *dSeipin* expression driven by the fat body-specific *Gal4*, *lsp2-Gal4* [31], rescued the defects in the fat body but not in the salivary gland or in the gut (Figure 4A, 4C and Figure S3). In contrast, *dSeipin* expression driven by a salivary gland-specific *Gal4*, *sgs3-Gal4* (which is expressed at late L3 stage) [32], fully rescued the ectopic lipid droplet phenotype in the salivary gland but not the lipid storage defects in the fat body or the ectopic lipid storage in the gut (Figure 4A, 4C and Figure S3). We were unable to obtain a suitable proventriculus- or anterior midgut-specific *Gal4* line for tissue-specific rescue. Nevertheless, these results indicate that the ectopic lipid droplets in the salivary gland and the midgut and the lipid storage defects in the fat body of *dSeipin* mutants are likely due to tissue-autonomous requirements of *dSeipin*. Furthermore, it implies that reduced lipogenesis in the fat body of *dSeipin* mutants did not lead to ectopic lipid storage in the salivary gland.

We further confirmed the tissue-autonomous requirements of *dSeipin* using a tissue-specific RNAi approach. The tissue-specific knockdowns of *dSeipin* were also confirmed by qRT-PCR (Figure S3). *UAS-dSeipin RNAi* driven by *ppl-Gal4* caused ectopic lipid storage in the salivary gland with mild lipid storage defects in the fat body, supporting the tissue-autonomous role of *dSeipin* (Figure 4B and 4C). However, *sgs3-Gal4*-driven *dSeipin* RNAi did not result in ectopic lipid storage in the salivary gland. We reasoned that *sgs3-Gal4* may act too late in the L3 larval stage to generate the RNAi effect. Indeed, *UAS-dSeipin RNAi* driven by either *48Y-Gal4* or *elav-Gal4*, which is expressed in salivary gland much earlier than *sgs3-Gal4* (Table S1), caused ectopic lipid droplet formation in the salivary gland (Figure 4B and 4C). Taking the data together, we concluded that *dSeipin* is required tissue-autonomously for preventing ectopic lipid droplet formation in salivary gland.

### *dSeipin* likely negatively affects lipid storage in salivary gland

The tissue-autonomous role of *dSeipin* prompted us to further explore the intrinsic mechanism of ectopic lipid droplet formation in *dSeipin* mutant salivary glands. Both increased lipogenesis and reduced lipolysis could theoretically result in lipid droplet formation. Which pathway is altered in *dSeipin* mutants and what is the function of *dSeipin* in that particular pathway?

**Figure 5:**
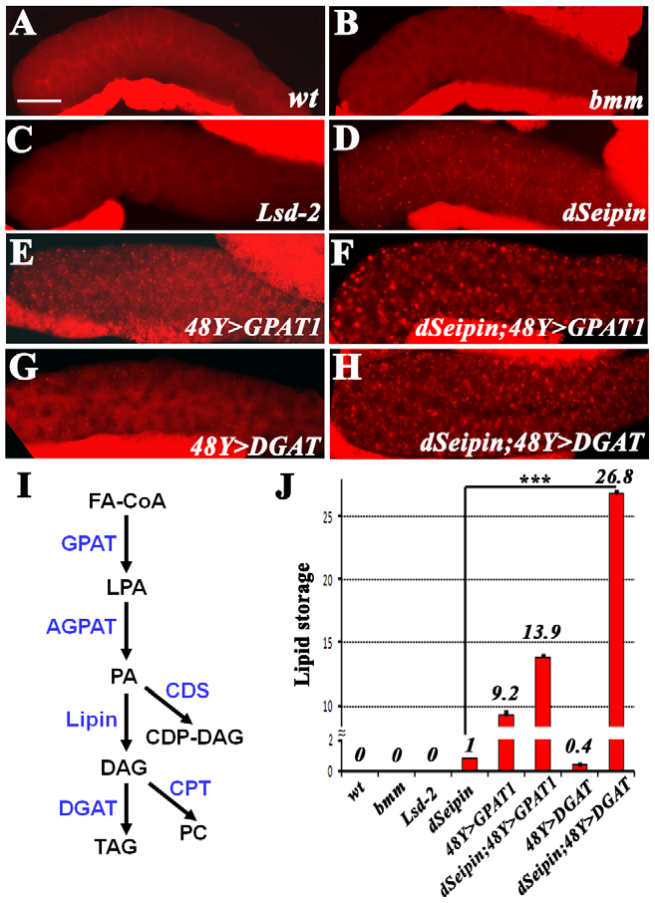
*dSeipin* genetically interacts with lipogenic genes. All images show Nile red staining of salivary glands. There are a few strongly stained fat body tissues next to the salivary glands. The genotypes are as follows: (A) Wild type, (B) *bmm^1^*, (C) *Lsd-2 ^KG00149^* (D) *dSeipin*, (E) *48Y>GPAT1*, (F) *dSeipin; 48Y>GPAT1*, (G) *48Y>DGAT*, and (H) *dSeipin; 48Y>DGAT*. *dSeipin* displays a strong synergistic genetic interaction with overexpression of *DGAT*. Scale bar: 100 µm. (I) The lipogenic pathway and the enzymes involved. (J) Quantification of (A–H). The error bars represent standard deviation. ***: P<0.0001.

*bmm* is a key positive regulator of lipolysis [28]. *bmm* loss-of-function mutants have reduced lipolysis, and display progressive obesity with increased synthesis of TAG, while overexpression of *bmm* results in a lean phenotype [21]. We found that *bmm* mutants have no ectopic lipid storage, suggesting that the ectopic lipid storage in salivary glands is not due to decreased lipolysis (Figure 5A and 5B). In addition, we found no ectopic lipid droplets in the salivary gland of *Lsd-2* mutants (Figure 5C), which also have a lean phenotype. Overexpression of *Lsd-2* with *ppl-Gal4* did not result in the ectopic lipid droplet phenotype either (data not shown). We next examined the lipogenic pathway. There are many enzymatic steps involved in the biosynthesis of TAG from fatty acids (Figure 5I) [33]. Firstly, fatty acids are converted to fatty acyl-CoA by acetyl-CoA synthetase (ACS). Fatty acyl-CoA has two different fates, either fatty acid oxidation to provide energy, or conversion to lysophosphatidic acid (LPA) by glycerol-3-phosphate acyltransferase (GPAT). Thus GPAT regulates the first committed step in lipogenesis and is likely a rate-limiting factor in lipogenesis [33], [34]. Acylglycerol phosphate acyltransferase (AGPAT) then adds another acyl chain to LPA to generate PA. PA can be converted to cytidine diphosphate diacylglycerol (CDP-DAG) by CDP diglyceride synthetase (CDS) or alternatively to DAG by Lipin, a PA phosphatase. CDP-DAG is the precursor of phosphatidylinositol (PI) and phosphatidylglycerol (PG). The last step in TAG biosynthesis, conversion of DAG to TAG, is catalyzed by DGAT. DAG can also be metabolized to phosphatidylcholine (PC) by choline phosphotransferase (CPT) or to phosphatidylethanolamine (PE) by ethanolamine phosphotransferase (EPT). All these enzymes are conserved in *Drosophila* (Table S2 and data not shown). We verified by qRT-PCR that all the corresponding genes are expressed in salivary gland (Figure S4). Since GPAT is the rate-limiting enzyme, we reasoned that overexpressing GPAT could lead to increased lipogenesis. Indeed, we found that overexpression of *GPAT1* (*CG5508*) using either *48Y-Gal4* or *ppl-Gal4* in wild-type flies caused an ectopic lipid droplet phenotype, indicating that increased lipogenesis can lead to ectopic lipid storage (Figure 5D, 5E and data not shown).

To examine whether *dSeipin* is involved in the lipogenesis pathway, we next analyzed the genetic interaction of *dSeipin* with *GPAT1* and *DGAT*, two key genes in the lipogenic pathway. Overexpression of *GPAT1* enhances the ectopic lipid storage phenotype of *dSeipin* mutants, consistent with the hypothesis that *dSeipin* mutants may have increased lipogenesis in non-adipose tissues (Figure 5D, 5E, 5F and 5J). Furthermore, although overexpression of *DGAT* using either *48Y-Gal4* or *ppl-Gal4* in wild type causes only mild lipid storage in salivary glands, overexpression of *DGAT* in a *dSeipin* mutant background results in a massive lipid storage phenotype in the salivary glands (Figure 5D, 5G, 5H, 5J and data not shown). Under these conditions, the salivary gland appears more like a lipid storage organ. We also verified the genetic interactions between *dSeipin* and *DGAT* with Bodipy, another lipid dye (Figure S4). Such strong synergistic genetic interactions in the metabolic pathway imply that in *dSeipin* mutants, DAG levels are likely increased in the salivary gland. Together, these results indicate that *dSeipin* probably negatively affects lipid storage in salivary gland.

### *dSeipin* may participate in the metabolism of PA in the lipogenic pathway

**Figure 6:**
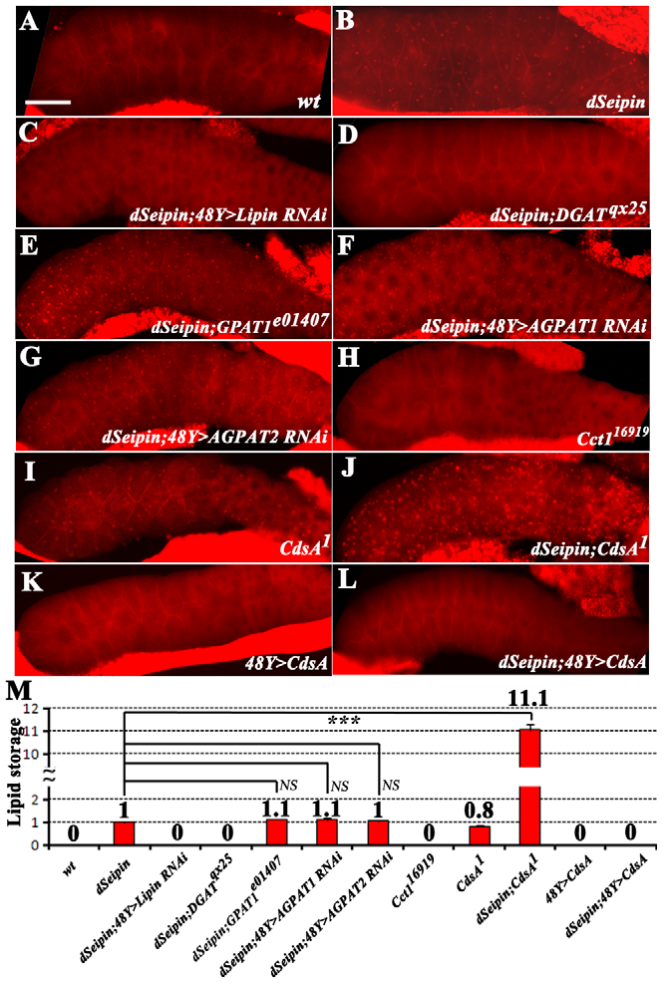
Seipin may affect the metabolism of PA. All images show Nile red staining of salivary glands. There are a few strongly stained fat body tissues next to the salivary glands. The genotypes are as follows: (A) wild type, (B) *dSeipin*, (C) *dSeipin;48Y>Lipin RNAi*, (D) *dSeipin;DGAT^qx25^*, (E) *dSeipin;GPAT1^e01407^*, (F) *dSeipin;48Y>AGPAT1 RNAi*, (G) *dSeipin;48Y>APGAT2 RNAi*, (H) *Cct1^16919^*, (I) *CdsA^1^*, (J) *dSeipin;CdsA^1^*, (K) *48Y>CdsA* and (L) *dSeipin;48Y>CdsA*. Ectopic lipid storage is present in B, E, F, G, I, and J. Scale bar: 100 µm. (M) Quantification of (A–L). *dSeipin* displays a synergistic interaction with *CdsA^1^*. The error bars represent standard deviation. NS: non-significant. ***: P<0.0001.

We next investigated at which point *dSeipin* acts in the lipogenesis pathway (Figure 5I). We examined mutants of *dSeipin* that also had loss-of-function mutations of the main lipogenic genes including *GPAT*, *AGPAT*, *Lipin*, and *DGAT*. We found that the ectopic lipid storage phenotype in *dSeipin* mutants was fully suppressed by a *DGAT* mutation or by RNAi of either *DGAT* or *Lipin* using either *48Y-Gal4* or *ppl-Gal4* (Figure 6A, 6B, 6C, 6D, 6M and data not shown). These results indicate that *dSeipin* may act upstream of *Lipin* and *DGAT*. In contrast, a partial loss-of-function mutant of *GPAT1* and RNAi of either *AGPAT1* or *AGPAT2*, two *AGPAT* homologs, could not suppress the *dSeipin* phenotype (Figure 6E, 6F, 6G, 6M and data not shown). Simultaneous RNAi of *AGPAT1* and *AGPAT2* also failed to suppress the *dSeipin* phenotype (data not shown). Due to the gene redundancy of *GPAT* and *AGPAT*, these results can't pinpoint the specific interaction between *dSeipin* and *GPAT1* or *AGPAT*. It is possible that *dSeipin* may not interact with *GPAT1* and *AGPAT*, or alternatively, *dSeipin* may act downstream of *GPAT1* and *AGPAT*. Since the connection point between *AGPAT* and *Lipin* is PA (Figure 5I), it is possible that *dSeipin* may affect the metabolism of PA so that *dSeipin* mutants have altered levels of PA, which subsequently leads to increased DAG and lipid storage.

To test our hypothesis genetically, we examined the salivary glands of two other mutants. *Cct1* is the rate limiting enzyme in PC biosynthesis, and loss of function of *Cct1* may lead to increased DAG (Figure 5I). *Cct1* RNAi was found to produce large lipid droplets in the S2 cell line [35] and *Cct1* mutants contain large lipid droplets in the fat body (data not shown). However, the salivary glands of *Cct1^16919^* mutants do not show ectopic lipid storage, suggesting that the *Cct1^16919^* mutation alone is insufficient to cause ectopic lipid droplets (Figure 6H and 6M). CdsA is the sole *Drosophila* homolog of human CDS and a partial loss-of-function *CdsA^1^* mutant has increased levels of a single species of PA (PA 16∶0/18∶2, also called PA 34∶2) (Figure 5I) [36]. In contrast to *Cct1^16919^* mutants, we found that *Drosophila CdsA^1^* mutants exhibit a similar ectopic lipid droplet phenotype to that of *dSeipin* mutants (Figure 6I and 6M). Therefore, we conclude that *dSeipin* may participate in PA metabolism.

We further checked the relationship between *dSeipin* and *CdsA* through double mutant analysis. *CdsA^1^;dSeipin* double mutants exhibit a strong synergistic phenotype compared to either single mutant (Figure 6J and 6M and Figure S4), indicating that *dSeipin* may function in parallel with *CdsA*. However, since *CdsA^1^* is a weak loss-of-function mutant, it is still possible that *dSeipin* functions in the same pathway as *CdsA*. In addition, overexpressing CdsA fully suppressed the ectopic lipid storage phenotype of *dSeipin* mutants (Figure 6K, 6L and 6M). Moreover, in *Drosophila* S2 cells, *dSeipin* also synergizes with *CdsA* in the formation of large lipid droplets (Figure S4). Consistent with the *dSeipin* RNAi result, *UAS-CdsA RNAi* driven by *48Y-Gal4* or *elav-Gal4*, but not *sgs3-Gal4*, resulted in ectopic lipid storage in the salivary gland, reflecting a tissue-autonomous role of CdsA and a tissue-autonomous mechanism of ectopic lipid droplet formation (data not shown). Taken together, these results suggest that *dSeipin* may be involved in the metabolism of PA and alteration of PA levels in *dSeipin* mutants may contribute to lipid droplets in salivary glands.

### PA and DAG levels are increased in *dSeipin* mutants

**Figure 7:**
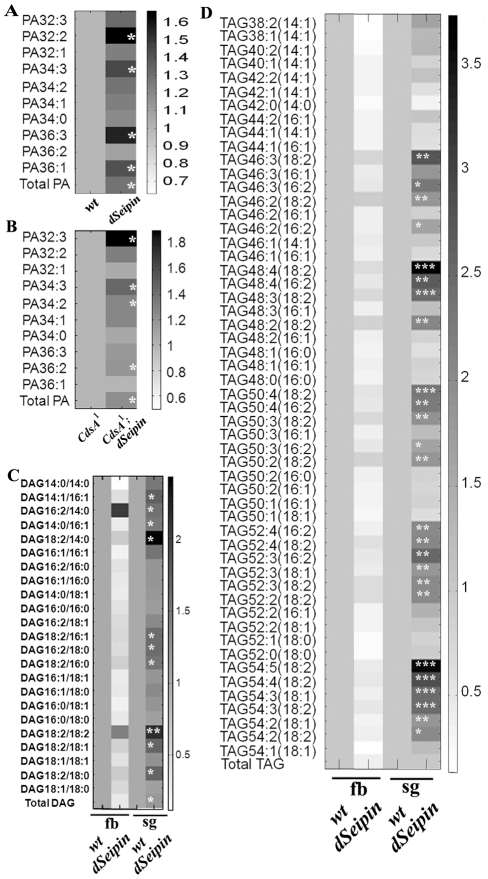
PA levels are increased in *dSeipin* mutants. (A) Heat plot showing relative levels of individual PA species and total PA species in *dSeipin* mutant larvae compared to wild type. The levels of total PA and most PA species are increased in *dSeipin* mutant larvae. *: P<0.05. (B) Heat plot showing relative levels of individual PA species and total PA species in the salivary glands of *CdsA^1^* mutants compared to *CdsA^1^;dSeipin* double mutants. There are more PA in *CdsA^1^;dSeipin* double mutants than *CdsA^1^* single mutants. *: P<0.05. (C) Heat plot showing relative levels of individual DAG species and total DAG species in the fat body (fb) and the salivary gland (sg) of *dSeipin* mutants compared to wild type. *: P<0.05; **: P<0.005. The levels of certain species of DAG are increased in the salivary gland of *dSeipin* mutants. (D) Heat plot showing relative levels of individual TAG species and total TAG species in *dSeipin* mutants compared to wild type. *: P<0.05; **: P<0.005; ***: P<0.0005. TAG levels are greatly reduced in the fat body (fb) of *dSeipin* mutants compared to wild type. The levels of many TAG species are increased in the salivary gland (sg) of *dSeipin* mutants compared to wild type.

To directly analyze whether PA levels are indeed increased in *dSeipin* mutants, we performed a lipidomic analysis of *dSeipin* mutants and wild type. We found that in *dSeipin* mutant larvae the levels of total PA and most PA species are increased (including PA32∶2, PA34∶3, PA36∶3, PA36∶1) (Figure 7A). To further confirm the genetic interaction results, we also compared PA levels in the salivary glands of *CdsA^1^* and *CdsA^1^;dSeipin* double mutants and found that there was more PA in *CdsA^1^;dSeipin* double mutants than *CdsA^1^* single mutants (Figure 7B). These results clearly demonstrate a role for dSeipin in the metabolism of PA.

In addition, the levels of total DAG and several DAG species, including 14∶0/18∶2, 16∶1/18∶2, 18∶ 0/18∶2, 18∶1/18∶2 and 18∶2/18∶2, are all significantly increased in mutant salivary glands (Figure 7C). These results are consistent with the strong genetic interaction between *dSeipin* and *DGAT* (Figure 5G, 5H and 5J). We also compared salivary gland TAG levels in different mutant backgrounds. In *dSeipin* mutants, the level of TAG is greatly reduced in the fat body, while the levels of several TAG species, including 46∶3(18∶2), 48∶4(18∶2), 50∶4(18∶2), 54∶4(18∶2), 54∶5(18∶2) are significantly increased in mutant salivary glands (Figure 7D). These results are consistent with the results obtained by Nile red staining (Figure 1). Similarly, we found that the TAG levels in *dSeipin;48Y>DGAT* and *dSeipin;CdsA^1^* double mutants are higher than *48Y>DGAT* or *CdsA^1^* alone (Figure S5). Together, these results support the conclusions obtained from the genetic analysis.

### *dSeipin* may have distinct functions in fat body and salivary gland

**Figure 8:**
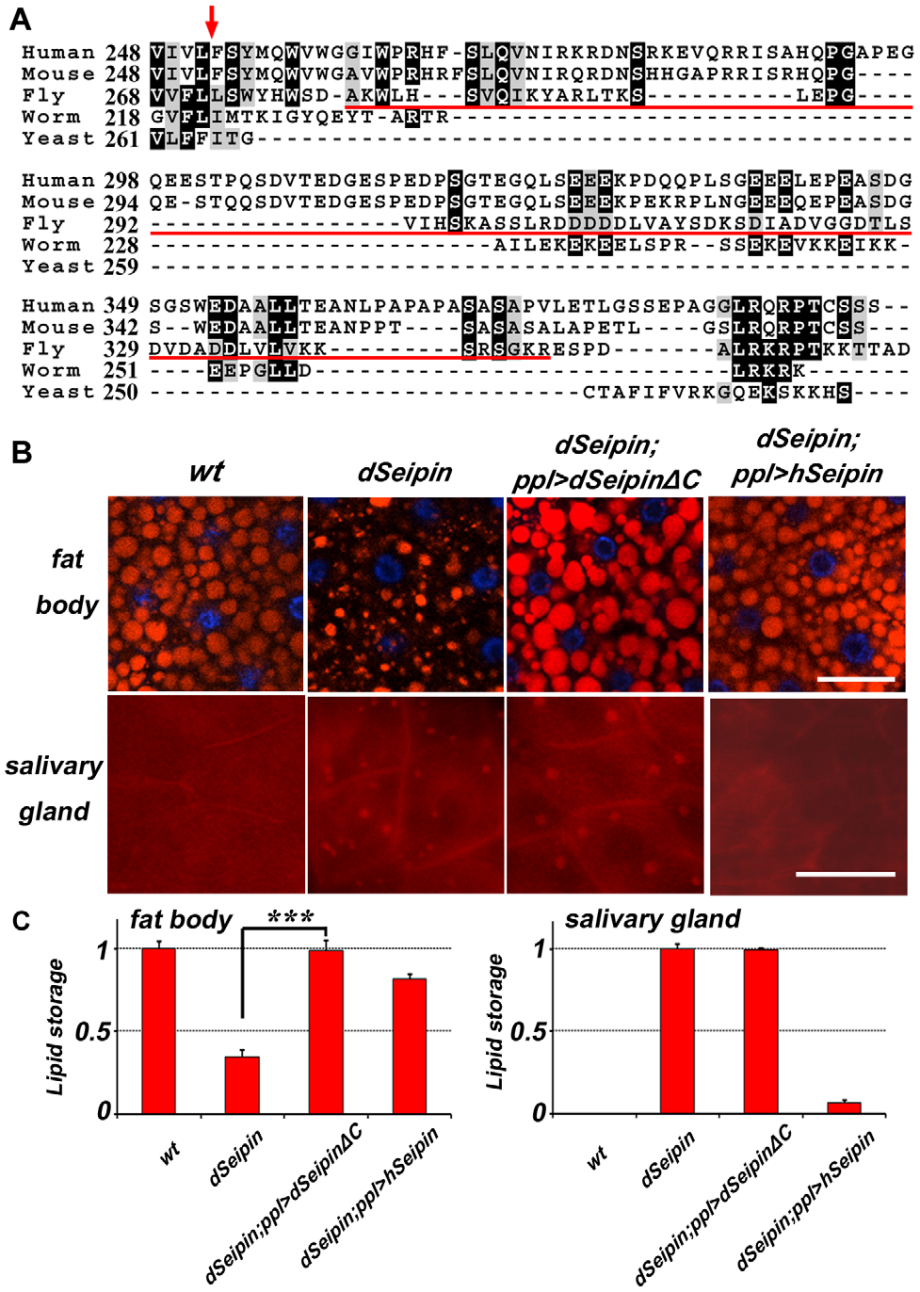
*dSeipin* may have distinct functions in different tissues. (A) Sequence alignment of the C-termini of Seipin homolog proteins from yeast (FLD1p), worm (R01B10.6), fly (CG9904), mouse (Q9Z2E9.2) and human (NP116056). The arrow points to the end of the second putative transmembrane domain. The region deleted in the C-terminal truncation of dSeipin is underlined (A280 to R352). (B) Red: Nile red staining; blue: DAPI staining. Expression of the C-terminal truncation of dSeipin in *dSeipin* mutants rescues the reduced lipid storage phenotype in the fat body but not the ectopic lipid storage phenotype in the salivary gland. Human Seipin (hSeipin) rescues the *dSeipin* mutant defects in both the fat body and the salivary gland. Scale bar: 50 µm. (C) Quantification of (B). The error bars represent standard deviation. ***: P<0.0001.

The above results indicate that *dSeipin* mutants have reduced lipid storage in the fat body but increased formation of lipid droplets in the salivary gland. Since *dSeipin* functions tissue autonomously, how could its absence cause opposite phenotypes in these two tissues? It is possible that there are distinct functions of *dSeipin* in different tissues. Alternatively, *dSeipin* may have the same function, but different tissues might respond differently to the same alteration of lipid contents. We reasoned that if dSeipin has distinct functions in different tissues, it might have some structural differences to yeast Seipin, because yeast is a unicellular organism. We noticed that both dSeipin and hSeipin have an extended C-terminal region compared to yeast Seipin (Figure 8A). Does the structure difference between dSeipin and yeast Seipin reflect the different functional requirement in various tissues in flies? To test this, we made a transgene with a C-terminal truncation of dSeipin and examined its rescuing activity in the fat body and the salivary gland of *dSeipin* mutants. This transgene, when driven by *ppl-Gal4*, fully rescued the reduced lipid storage phenotype in the fat body, but not the ectopic lipid droplet phenotype in the salivary gland (Figure 8B and 8C). Although this result does not rule out the possibility that dSeipin functions similarly in different tissues, which respond differently to altered lipid levels, it more strongly suggests that dSeipin may have distinct functions in fat body and salivary gland. Moreover, it echoes previous conclusions that dSeipin functions tissue-autonomously.

### Seipin function is evolutionarily conserved

To test whether the function of Seipin is evolutionarily conserved, we expressed hSeipin in *dSeipin* mutants and examined its rescue effect. We found that hSeipin can functionally replace dSeipin in the fat body and the salivary gland. Lipid storage in the fat body is restored and ectopic lipid storage in the salivary gland is reversed in *dSeipin* mutants with *ppl-Gal4*-driven *UAS-hSeipin* (Figure 8B and 8C). N88S and S90L, two point mutations of hSeipin, were previously found to be associated with Silver syndrome, a dominant motor neuron degenerative disease [37]. The S90L hSeipin mutation also rescued the fat body and salivary gland phenotypes of *dSeipin* mutants, supporting the previous finding that S90L is likely a gain-of-function mutation (data not shown) [37]. These results indicate that the function of Seipin is conserved between fly and human.

## Discussion

BSCL2 is a severe form of lipodystrophy which affects adipocyte development and results in ectopic lipid storage in non-adipose tissues. The exact function of BSCL2/Seipin and the causes of ectopic lipid storage are not known. Here we report analyses of the *Drosophila dSeipin* mutant. *dSeipin* mutants have reduced lipid storage in the fat body, which is the *Drosophila* adipose tissue, and ectopic lipid droplets in the salivary gland, a non-adipose tissue. It is worth noting that to the best of our knowledge the ectopic lipid droplet phenotype has not been reported previously in any invertebrate model organism.

Consistent with previous findings that Seipin has a cell-autonomous function in the regulation of adipogenesis and adipocyte differentiation [13], [14], our results reveal a tissue-autonomous role of dSeipin in controlling lipid storage in adipocytes. Since the function of Seipin is conserved through evolution (this study and [18], [19]), mammalian Seipin may have a similar role in lipid storage in mature adipocytes. Moreover, although the gross morphology of *dSeipin* mutant fat bodies appears normal, the lipid storage defects may reflect impaired fat body differentiation.

More importantly, our studies uncover an unexpected tissue-autonomous role of Seipin in preventing ectopic lipid storage in non-adipose tissues. Ectopic lipid storage is one of the main causes of pathological conditions in obesity and lipodystrophy [4], [5]. Our in vivo studies demonstrated that defects within non-adipose tissues are the primary cause of ectopic lipid storage in *dSeipin* mutants. Is this tissue-autonomous mechanism specific to Seipin and lipodystrophy? Many mouse models of lipodystrophy and obesity associated with ectopic lipid storage in liver and muscles have been reported. High serum free fatty acid levels are thought to be the primary cause of liver steatosis in several studies, implicating tissue-non-autonomous regulatory effects in ectopic lipid storage [38], [39]. Interestingly, mice deficient in *AGPAT2*, the BSCL1 lipodystrophy gene, were recently found to have normal or low levels of serum free fatty acids, but still developed robust liver steatosis [40]. Instead of being caused primarily by high serum free fatty acid levels, hepatic steatosis in *AGPAT2* mice could be explained by a tissue-autonomous function of AGPAT2. If this is true, both AGPAT2 and Seipin probably have tissue-autonomous functions in preventing ectopic lipid storage. Thus, the tissue-autonomous mechanism of ectopic lipid storage could be a general theme in lipodystrophy. It will be interesting to examine whether the ectopic lipid storage in previously reported mouse models of obesity and lipodystrophy are caused by tissue-autonomous or tissue-non-autonomous mechanisms.

Within a cell, lipid storage could originate in two ways, increased lipogenesis and reduced lipolysis. We hypothesize that *Seipin* is involved in lipogenesis. The synergistic genetic interactions between *dSeipin* and lipogenic genes, in particular *DGAT*, strongly argue that *Seipin* participates in the lipogenic pathway. Ectopic lipid storage in salivary glands was observed in animals with overexpression of the lipogenic gene *GPAT1*, but not in loss-of-function mutants of the lipolytic gene *bmm*, suggesting that increased lipogenesis but not reduced lipolysis causes ectopic lipid storage in vivo. Thus, *Seipin* mutants may have increased lipogenic activity in non-adipose tissues, which subsequently results in the formation of ectopic lipid droplets in midgut and salivary glands as shown in Figure 3A and 3B.

Based on the lipidomic data and the genetic interactions between *dSeipin* and lipogenic genes, we propose that dSeipin participates in PA metabolism. Several lines of evidence support this hypothesis. Firstly, *CdsA^1^* mutants, which have increased levels of PA34∶2, exhibit the same ectopic lipid droplet phenotype as *dSeipin* mutants (Figure 6I). Secondly, *dSeipin* synergizes with *CdsA^1^*, which is a partial loss-of-function mutant of *CdsA*, in ectopic lipid droplet formation (Figure 6J). Thirdly, overexpression of CdsA can fully suppress the ectopic lipid droplet phenotype of *dSeipin* mutants (Figure 6L). Fourthly, *dSeipin* also synergizes with *CdsA* in the formation of large lipid droplets in *Drosophila* S2 cells (Figure S4). Lastly, *AGPAT2*-deficient mice have increased levels of PA, which may lead to ectopic lipid storage in a tissue-autonomous fashion [40]. Therefore, it is likely that three known lipodystrophy genes (*AGPAT2*, *Lipin* and *Seipin*) are all involved in PA metabolism [11], [12], [41]. Although it has been suggested that hepatic TAG accumulation in *AGPAT2*-deficient mice is caused by a bypass pathway from LPA to monoacylglyceride (MAG) and subsequently to TAG, the contribution of elevated PA to excess TAG remains to be determined. Moreover, the exact cause of the increased levels of PA in *AGPAT2*-deficient mice is unclear. It could be due to elevated DAG kinase activity or increased expression of other AGPATs, such as AGPAT1, 3, and 8 [40]. The lipid profile of human *Seipin* mutant lymphoblastoid cell lines has been studied [42]. However, in that study the levels of PA weren't measured. Interestingly, it was found that the levels of unsaturated TAG species are decreased along with the increases of saturated TAG species. In our lipidomic analysis, the levels of unsaturated DAG and TAG species were significantly increased in salivary gland (Figure 7). These results suggest that different cells/tissues may response differently to *Seipin* mutation.

Although the exact molecular function of dSeipin remains unclear, we propose that Seipin might act as an enzyme or a cofactor in regulating glycerolipid (likely PA) metabolism. PA occupies a specific branch point in the glycerolipid biosynthetic pathway. It can be converted to DAG by Lipin, or to CDP-DAG by CDS. DAG is the immediate precursor of TAG and PC, while CDP-DAG is the precursor of PI and PG. We propose that Seipin may influence lipogenesis by diverting PA from the lipogenic pathway in non-adipose tissues, such as the salivary gland. In the absence of Seipin the lipogenic pathway is more active, leading to lipid droplet formation. In addition, it is possible that PA could influence the formation of lipid droplets since the surface of lipid droplets is a monolayer of polar lipids.

*dSeipin* mutants display opposite phenotypes in the fat body and salivary gland, but we still do not know why there is reduced lipid storage in the fat body of *dSeipin* mutants. It is possible that compared to the salivary gland, the fat body responds differently to altered levels of phospholipids. Alternatively, Seipin might have different roles in different tissues. These two possibilities are not mutually exclusive, although the specific rescue of the lipid storage phenotype in the fat body but not the salivary gland by C-terminal truncation of dSeipin favors the latter possibility. Identification of the specific function and the binding partner of the C-terminal region in the near future will shed more light on the exact function of Seipin.

In summary, the *Drosophila Seipin* model has not only revealed a novel tissue-autonomous mechanism of ectopic lipid storage in lipodystrophy but has provided a new genetic tool to further identify the regulatory machinery controlling lipid storage. Additional studies combining yeast, *Drosophila* and mouse models will further advance our knowledge on lipodystrophy and benefit the development of therapeutic strategies to combat lipid storage diseases such as obesity.

## Materials and Methods

### *Drosophila* stocks and husbandry

Unless specified, *Drosophila* stocks were maintained in standard corn meal food with Angel dry yeast (Angel Yeast CO., LTD, Hubei, China). Canton-S was used as wild type and the transgenic line for generating *dSeipin* null mutants was treated as the control in some experiments where indicated. All stocks were obtained from the Bloomington Stock Center, the Harvard collection or the Vienna *Drosophila* RNAi center (for all RNAi stocks) except for *ppl-Gal4*, *bmm^1^*, *UAS-bmm*, and *CdsA^1^*. All *Gal4* lines were verified by crossing to *UAS-GFP* before use (Table S1). The effects of overexpression or RNAi of many lipogenic genes were also verified by qRT-PCR (Figure S4). To generated *dSeipin* null mutants, we followed the ends-out procedure developed by Golic et al [43] with minor modifications [44]. A *dSeipin* knockout allele was isolated and confirmed by PCR. The mutant was backcrossed three times to *w^1118^* (control) to eliminate background mutations. Transgenic stocks were generated by standard methods.

### Molecular biology

The coding region of *Lsd-1* (without the stop codon) was amplified by RT-PCR and inserted into a T vector. The *Lsd-1-mCherry* fusion was created by ligating the *Lsd-1* coding region (EcoRI-KpnI) in frame into a mCherry vector. The *Lsd-1-mCherry* fragment (EcoRI and XbaI) was shuttled to the transformation vector *pUAST-attB* to yield *UAS-Lsd-1-mCherry*. For *UAS-dSeipin*, full length cDNA was amplified from clone SD04409 and inserted into the *pUAST-attB* vector (EcoRI and XhoI). *UAS-dSeipin-YFP* was generated by replacing the stop codon of the cDNA with a BglII site. For *UAS-hSeipin*, *hSeipin* cDNA was amplified from human SY5Y cells and inserted into the BglII and XhoI sites of the *pUAST* vector. The S90L mutation of *hSeipin* was generated through site-directed mutagenesis. All constructs requiring PCR amplification were confirmed by sequencing. All qRT–PCRs were performed on an ABI PRISM 7900HT real-time cycler (Applied Biosystems) using Power SYBR Green PCR Master Mix (Applied Biosystems). Primer sequences are listed in Table S3.

### Staining and microscopy

For in situ hybridization, a 270bp *dSeipin* cDNA fragment was amplified and subcloned into *pEasy-T3* vector with the following primers: 5′-agatctATGCCGGCCATATCGCACAC-3′and 5′-aagcttGCGCATCATGGCAGACCGAC-3′. An anti-sense digoxygenin-labeled probe was made using a BglII-linearized template. Hybridization was detected by using anti-DIG alkaline phosphatase and the CBIP/NBT substrate (Roche). For lipid droplet staining, larvae were dissected in PBS and fixed in 4% paraformaldehyde/PBS for 30 min at room temperature. Tissues were then rinsed twice with 1×PBS, incubated for 30 min in either a 1∶2500 dilution with PBS of 0.5mg/ml Nile red (Sigma), or 0.06% Oil Red O (Sigma), or a 1∶1000 dilution with PBS of 1mg/ml BODIPY 493/503 (Invitrogen), and then rinsed twice with distilled water. Stained samples were mounted in 80% glycerol. For lysotracker staining, fed or starved larvae were dissected in 1∶1000 lysotracker (Invitrogen) and incubated for 5 min before mounting to a slide. All images were taken using a Nikon confocal scope or Zeiss fluorescent scope. The relative levels of lipid storage in fat body cells and salivary glands were quantified separately. Briefly, for lipid storage in fat body cells, the Nile red-positive areas of 30 fat body cells per genotype were measured by NIS-Elements BR 3.0 and then normalized to the whole cell area. The average lipid storage in wild type was set as 1. For lipid storage in salivary glands, the Nile red positive areas of 15 salivary glands per genotype were measured by NIS-Elements BR 3.0 and normalized to the whole cell area. The average lipid storage in *dSeipin* mutants was set as 1.

### Cell culture and RNAi by dsRNA soaking

*Drosophila* S2 cells were cultured in Schneider's *Drosophila* medium (Invitrogen) supplemented with 10% fetal bovine serum (FBS) at 25°C. dsRNA for RNAi treatment was produced by in vitro transcription of a PCR-generated DNA template containing the T7 sequence at both ends. The dsRNAs were generated using a MEGAscript T7 kit (Ambion). Two different sets of primers were used for targeted genes, and the one with better RNAi efficiency was used for the experiments reported. The primer sequences for *dSeipin* were: forward, 5′-gaattaatacgactcactatagggagaCCATATCGCACACCCGAC-3′, and reverse, 5′-gaattaatacgactcactatagggagaACTATGGCCGACAATACG-3′. The primer sequences for *CdsA* were: forward, 5′-gaattaatacgactcactatagggagaTTTGGTTCGTGCTCTCACTG-3′, and reverse, 5′-gaattaatacgactcactatagggagaCGTGAACAAATAGCTTGGCA-3′. S2 cells were diluted to a final concentration of 5×10^6^ cells/ml in Schneider's *Drosophila* medium without FBS in 6-well plates. 20 µg dsRNA was added to 1 ml of cell suspension and incubated for 45 minutes at 25°C. After the incubation, 3 ml complete medium was added and the cells were cultured for another 3 days. Cells were collected and split into two for total RNA extraction and Bodipy staining.

### *Drosophila* phenotypic analysis

For larvae starvation, wild type and mutant embryos were collected within a 4 hr period and raised at low density on standard fly food at 25°C. 60–65 hr after hatching, larvae were either fed with normal food or starved in PBS for 24 hr. Fed and starved larvae were then dissected and stained with Nile red or Oil Red O. Adult starvation tests were performed by transferring flies into vials (25 flies per vial) with filter papers soaked with distilled water. Mortality rates were determined by regularly counting the number of dead flies. For each genotype, triplicate batches of 75 male flies each (<36 hr of age) were used.

### Glyceride analysis

To determine total glyceride, ten male flies were homogenized in 100 µl PBST (0.05% Tween 20), incubated at 70°C for 15 min and then centrifuged at 1,200 rpm for 5 min. The supernatants were incubated with Triglyceride analysis reagent (Biosino Biotechnology) at 37°C for 10 min before being analyzed with a Bio-RAD 550 microplate reader at 490 nm. Glyceride levels were normalized to protein levels using a Bradford assay. Hemolymph was collected from L3 larvae (20 from each group) and diluted in 50 µl PBST (0.05% Tween 20), heated at 70°C for 5 min, and centrifuged at 12,000 rpm for 5 min. Glyceride in the hemolymph supernatant was measured using TAG determination kits (Sigma).

### Analysis of lipids using high performance liquid chromatography/mass spectrometry

Lipids from salivary glands of 50 larvae, fat bodies of 50 larvae or 10 whole larvae (three sets of samples per genotype) were extracted as previously described [36]. An Agilent high performance liquid chromatography (HPLC) system coupled with an Applied Biosystem Triple Quadrupole/Ion Trap mass spectrometer (3200Qtrap) was used for quantification of individual phospholipids. Multiple reaction monitoring (MRM) transitions were set up for quantitative analysis of various polar lipids [19], [45]. Normal phase HPLC was set up for separation of individual lipid classes. Levels of individual lipids were quantified using spiked internal standards including dimyristoyl dimyristoyl phosphatidic acid (28∶0-PA), which was obtained from Avanti Polar Lipids. Neutral lipids were analyzed using a sensitive HPLC/ESI/MRM method modified from a previous method [46]. TAG levels were calculated relative to the spiked d5-TAG 48∶0 internal standard (CDN Isotopes Inc.), while DAGs were quantified using 4ME 16∶0 Diether DG (Avanti) as an internal standard. The results from three experiments were normalized and plotted in a heat map.

## Supporting Information

Figure S1 The Seipin homolog in *Drosophila*. (A) Sequence alignment of Seipin homolog proteins from yeast (FLD1p), worm (R01B10.6), fly (CG9904), mouse (Q9Z2E9.2) and human (NP116056). Human Seipin has two isoforms and only the short isoform (NP116056) was used for alignment. The two residues (N88 and S90) in hSeipin that are mutated in Silver syndrome are marked with asterisks. (B) The expression pattern of *dSeipin* mRNA is revealed by in situ hybridization. Black arrow: hindgut in the embryo; white arrow: nuclei of larval fat body; arrowhead: larval anterior midgut. *dSeipin* mutants were used as negative controls. (C) Colocalization of dSeipin-YFP with the ER marker PDI-GFP. To detect YFP signal and minimize GFP fluorescent leak-through, the emission detector in the confocal microscope was set up to allow only strong YFP signal to pass.
(11.18 MB TIF)

Figure S2 Phenotypic analysis of *dSeipin* mutants. (A) Autophagy in fat bodies labeled by lysotracker under starved and fed conditions. Wild type and *dSeipin* mutants exhibit positive punctate lysotracker staining only under starved conditions. (B) Reduced lipid storage in the wing disc of *dSeipin* mutants. Scale bar: 100 µm (C–E) *dSeipin* mutants show no ectopic lipid storage in the brain (C, scale bar: 100 µm), muscle (D, scale bar: 50 µm) and epidermis (E, scale bar: 100 µm). B, D and E are Oil Red O staining; C is Nile red staining.
(9.94 MB TIF)

Figure S3 Tissue-specific function of dSeipin. (A–D) qRT-PCR analysis of *dSeipin* transcription in various genetic backgrounds. Transcription level of *dSeipin* in fat bodies (A) and salivary glands (B) of tissue specifically-rescued *dSeipin* mutants were examined. (C) and (D) show the transcription level of *dSeipin* in fat bodies and salivary glands of tissue-specific *dSeipin* RNAi animals. (E) The ectopic lipid storage phenotype in the midgut of *dSeipin* mutants cannot be rescued by fat body-specific or salivary gland-specific expression of dSeipin. Red: Oil Red O Staining. Scale bar: 100 µm. (F) Oenocyte-specific expression of dSeipin cannot restore oenocyte lipid storage under starvation conditions in *dSeipin* mutants.
(5.69 MB TIF)

Figure S4 *dSeipin* genetically interacts with *DGAT* and *CdsA*. (A) qRT-PCR analysis of the transcription of lipid metabolism-related genes in salivary glands. *rp49* was used as control. (B) qRT-PCR results of the transcription of lipid metabolism-related genes in various genetic backgrounds. EP or UAS lines were used for gene overexpression. (C) Bodipy staining of salivary glands in various genetic backgrounds. There are a few strongly stained fat body tissues next to the salivary glands. The genotypes are as indicated. *dSeipin* displays synergistic interactions with *CdsA^1^* and overexpression of *DGAT*. (D) Synergistic interaction of *dSeipin* and *CdsA* in S2 cells. The cells were treated with dsRNA and stained with Bodipy. The efficiency of RNAi is indicated by RT-PCR. (E) The size and the number of lipid droplets (LD) in the RNAi experiment (D) were quantified. For the analysis, results from 35 random cells were pooled and graphed in scattered plot. Each data point represents an individual lipid droplet. The relative size of lipid droplets was increased in *dSeipin* dsRNA-treated cells. CdsA dsRNA treatment further increases the LD size, indicating the synergistic interaction of *dSeipin* and *CdsA*. NS: non-significant. ***: P<0.001.
(6.70 MB TIF)

Figure S5 TAG levels in different genetic backgrounds. Heat plot showing relative levels of individual TAG species and total TAG species in different genetic backgrounds. *: P<0.05; **: P<0.005; ***: P<0.0005. (A) In the salivary gland, *dSeipin* mutation increases the levels of TAG in animals which overexpress DGAT (*48Y>DGAT*). (B) In the salivary gland, *dSeipin* mutation increases the levels of TAG in *CdsA^1^* mutants.
(4.03 MB TIF)

Table S1 Expression patterns of the *Gal4* lines used in this study.
(0.03 MB DOC)

Table S2 Lipid metabolism-related genes and alleles used this study.
(0.05 MB DOC)

Table S3 Primers for qRT-PCR.
(0.04 MB DOC)
