# Supplementary figures and images for: Tissue-Autonomous Function of Drosophila Seipin in Preventing Ectopic Lipid Droplet Formation
Source: PLoS Genet. 2011 Apr 14;7(4):e1001364. doi: 10.1371/journal.pgen.1001364 (PMC3077376; doi:10.1371/journal.pgen.1001364)

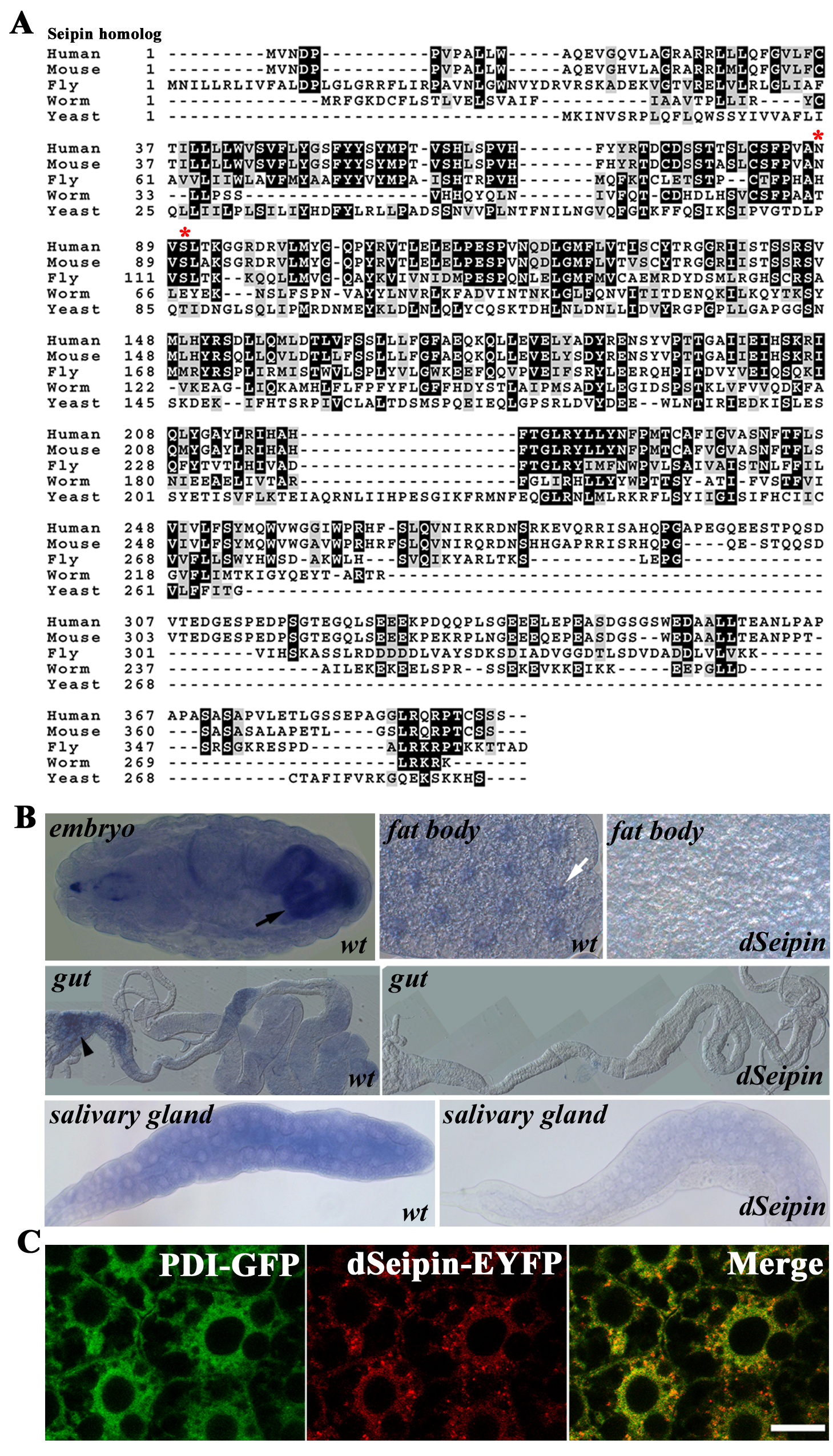

Supplement: Figure S1 — The Seipin homolog in Drosophila. (A) Sequence alignment of Seipin homolog proteins from yeast (FLD1p), worm (R01B10.6), fly (CG9904), mouse (Q9Z2E9.2) and human (NP116056). Human Seipin has two isoforms and only the short isoform (NP116056) was used for alignment. The two residues (N88 and S90) in hSeipin that are mutated in Silver syndrome are marked with asterisks. (B) The expression pattern of dSeipin mRNA is revealed by in situ hybridization. Black arrow: hindgut in the embryo; white arrow: nuclei of larval fat body; arrowhead: larval anterior midgut. dSeipin mutants were used as negative controls. (C) Colocalization of dSeipin-YFP with the ER marker PDI-GFP. To detect YFP signal and minimize GFP fluorescent leak-through, the emission detector in the confocal microscope was set up to allow only strong YFP signal to pass. (11.18 MB TIF) [file pgen.1001364.s001.tif]

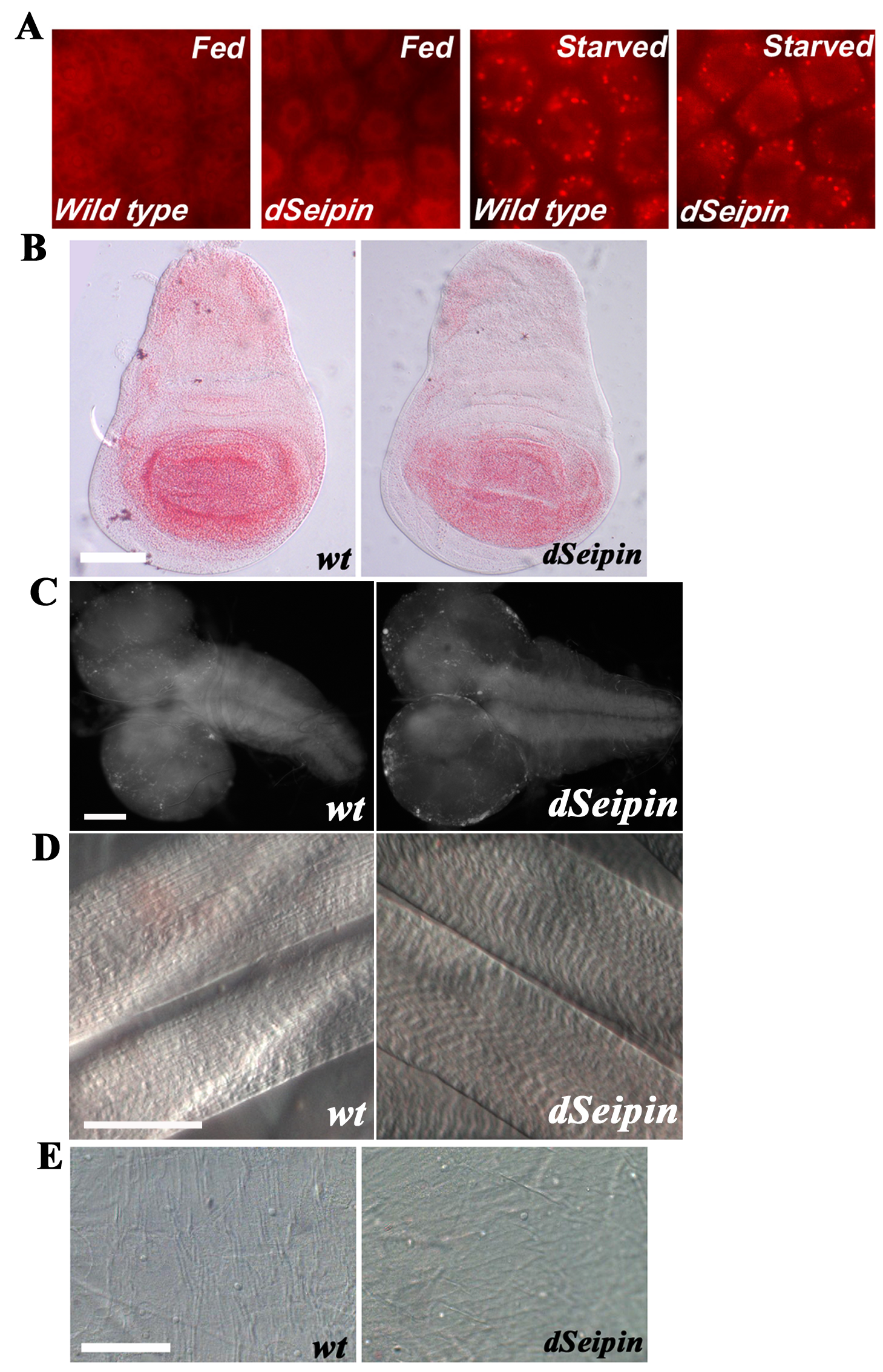

Supplement: Figure S2 — Phenotypic analysis of dSeipin mutants. (A) Autophagy in fat bodies labeled by lysotracker under starved and fed conditions. Wild type and dSeipin mutants exhibit positive punctate lysotracker staining only under starved conditions. (B) Reduced lipid storage in the wing disc of dSeipin mutants. Scale bar: 100 µm (C–E) dSeipin mutants show no ectopic lipid storage in the brain (C, scale bar: 100 µm), muscle (D, scale bar: 50 µm) and epidermis (E, scale bar: 100 µm). B, D and E are Oil Red O staining; C is Nile red staining. (9.94 MB TIF) [file pgen.1001364.s002.tif]

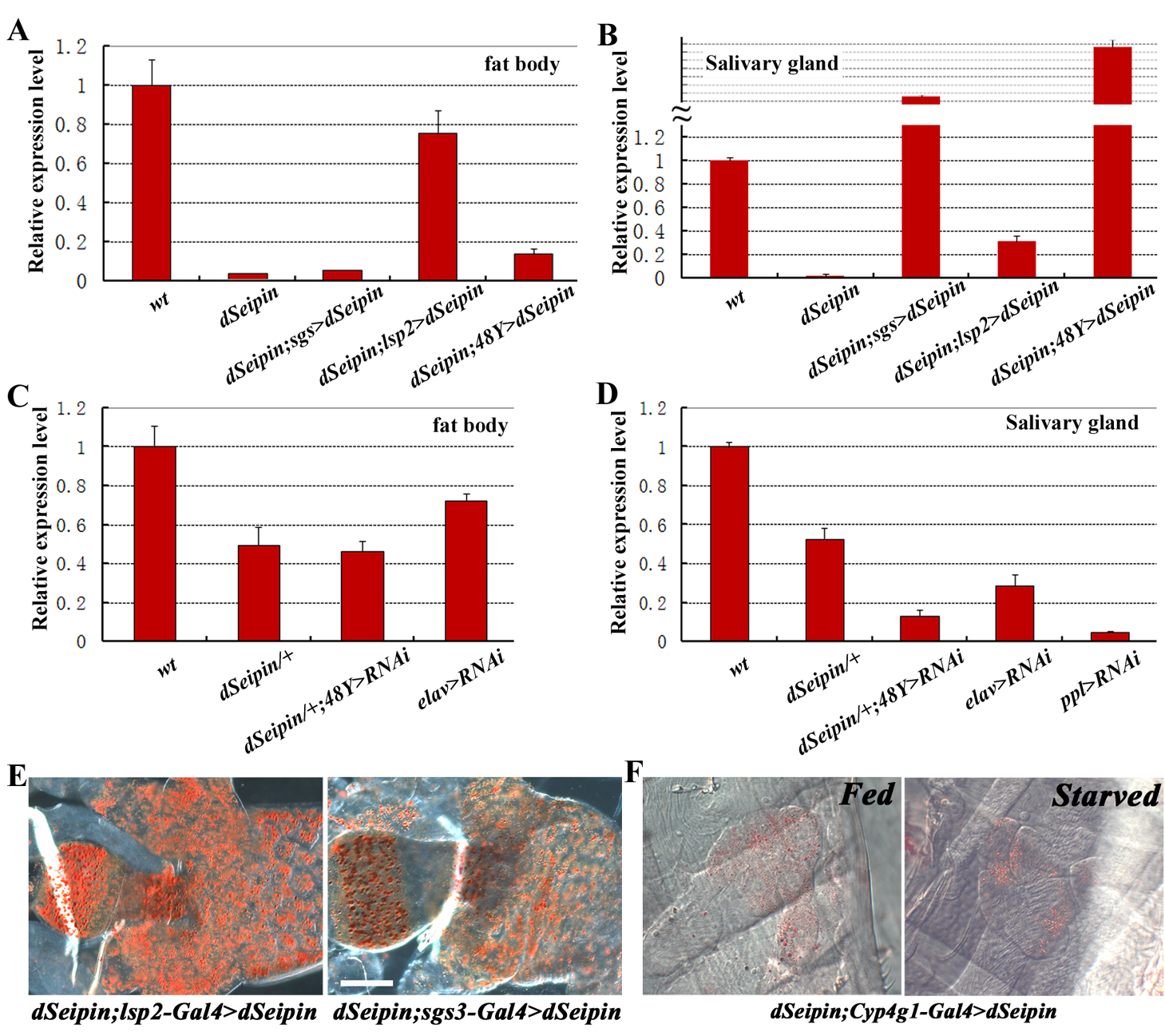

Supplement: Figure S3 — Tissue-specific function of dSeipin. (A–D) qRT-PCR analysis of dSeipin transcription in various genetic backgrounds. Transcription level of dSeipin in fat bodies (A) and salivary glands (B) of tissue specifically-rescued dSeipin mutants were examined. (C) and (D) show the transcription level of dSeipin in fat bodies and salivary glands of tissue-specific dSeipin RNAi animals. (E) The ectopic lipid storage phenotype in the midgut of dSeipin mutants cannot be rescued by fat body-specific or salivary gland-specific expression of dSeipin. Red: Oil Red O Staining. Scale bar: 100 µm. (F) Oenocyte-specific expression of dSeipin cannot restore oenocyte lipid storage under starvation conditions in dSeipin mutants. (5.69 MB TIF) [file pgen.1001364.s003.tif]

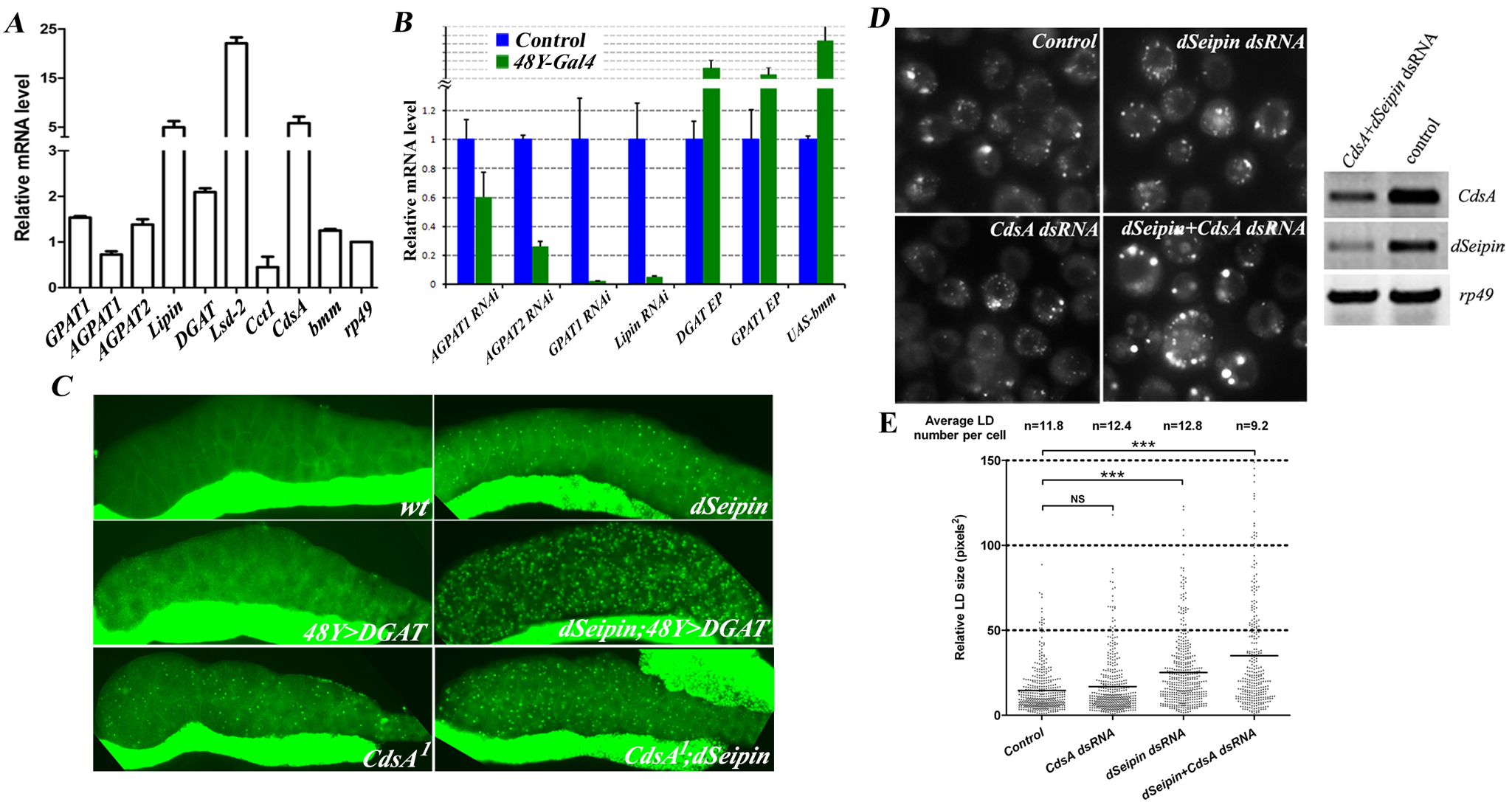

Supplement: Figure S4 — dSeipin genetically interacts with DGAT and CdsA. (A) qRT-PCR analysis of the transcription of lipid metabolism-related genes in salivary glands. rp49 was used as control. (B) qRT-PCR results of the transcription of lipid metabolism-related genes in various genetic backgrounds. EP or UAS lines were used for gene overexpression. (C) Bodipy staining of salivary glands in various genetic backgrounds. There are a few strongly stained fat body tissues next to the salivary glands. The genotypes are as indicated. dSeipin displays synergistic interactions with CdsA1 and overexpression of DGAT. (D) Synergistic interaction of dSeipin and CdsA in S2 cells. The cells were treated with dsRNA and stained with Bodipy. The efficiency of RNAi is indicated by RT-PCR. (E) The size and the number of lipid droplets (LD) in the RNAi experiment (D) were quantified. For the analysis, results from 35 random cells were pooled and graphed in scattered plot. Each data point represents an individual lipid droplet. The relative size of lipid droplets was increased in dSeipin dsRNA-treated cells. CdsA dsRNA treatment further increases the LD size, indicating the synergistic interaction of dSeipin and CdsA. NS: non-significant. ***: P<0.001. (6.70 MB TIF) [file pgen.1001364.s004.tif]

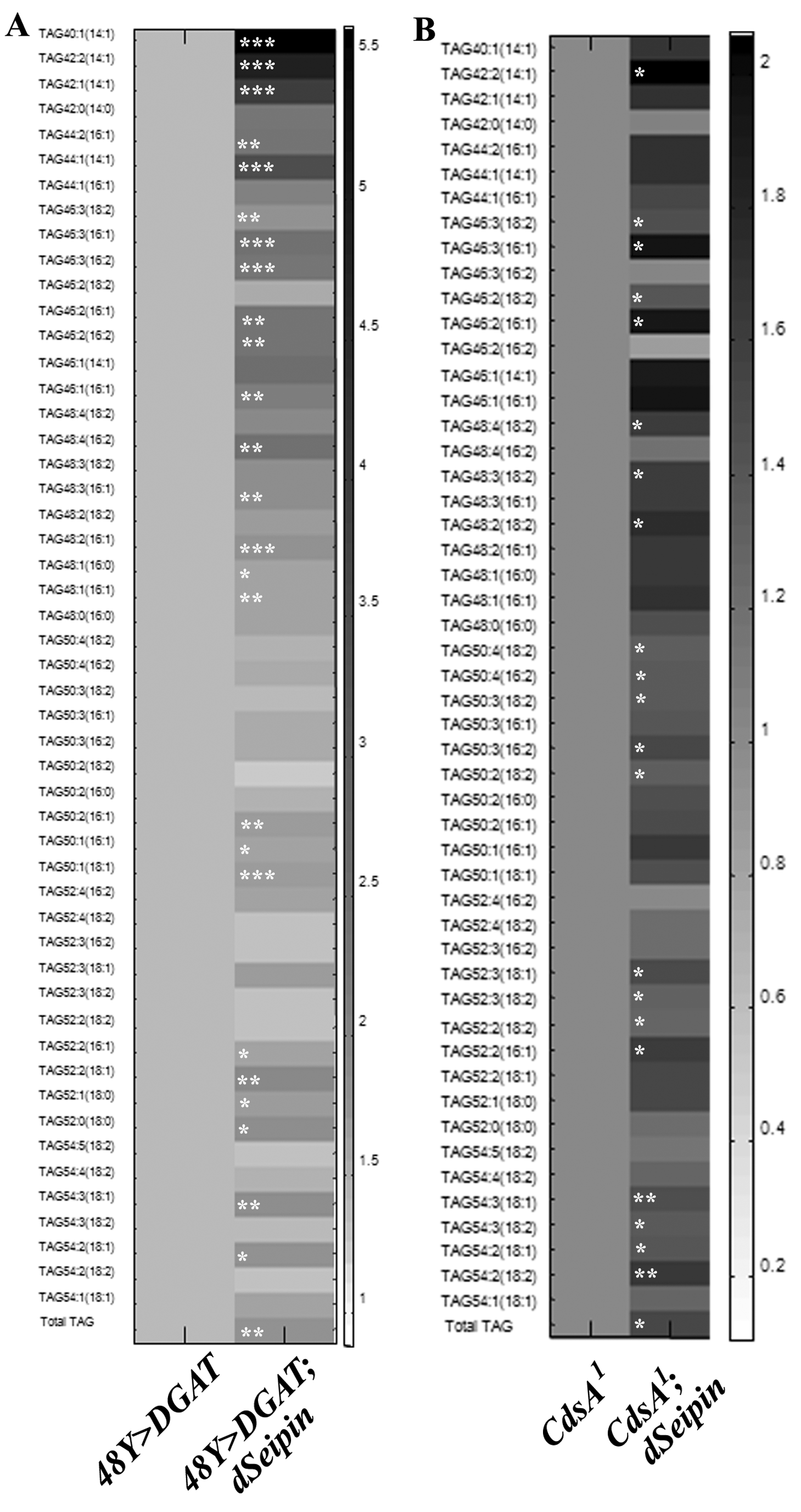

Supplement: Figure S5 — TAG levels in different genetic backgrounds. Heat plot showing relative levels of individual TAG species and total TAG species in different genetic backgrounds. *: P<0.05; **: P<0.005; ***: P<0.0005. (A) In the salivary gland, dSeipin mutation increases the levels of TAG in animals which overexpress DGAT (48Y>DGAT). (B) In the salivary gland, dSeipin mutation increases the levels of TAG in CdsA1 mutants. (4.03 MB TIF) [file pgen.1001364.s005.tif]
