# Supplementary material for: Tissue-Autonomous Function of Drosophila Seipin in Preventing Ectopic Lipid Droplet Formation
Source: PLoS Genet. 2011 Apr 14;7(4):e1001364. doi: 10.1371/journal.pgen.1001364 (PMC3077376; doi:10.1371/journal.pgen.1001364)
Supplement: Table S1 — Expression patterns of the Gal4 lines used in this study. (0.03 MB DOC) [file pgen.1001364.s006.doc]

**Supporting Table1.** Expression patterns of the *Gal4* lines used in this study

| ***Gal4*** | **Expressed in:** | **Not expressed in:** |
| --- | --- | --- |
| *ppl-Gal4* | salivary gland (strong), fat body |  |
| *lsp2-Gal4* | fat body | salivary gland |
| *sgs3-Gal4* | salivary gland (late L3 larval stage) | fat body |
| *48Y-Gal4* | embryonic endoderm, salivary gland | fat body |
| *elav-Gal4* | salivary gland and nervous system | fat body |
| *Cyp4g1-Gal4* | oenocytes | fat body |

The expression patterns were verified by crossing to a *UAS-GFP* line.
