# Supplementary material for: Tissue-Autonomous Function of Drosophila Seipin in Preventing Ectopic Lipid Droplet Formation
Source: PLoS Genet. 2011 Apr 14;7(4):e1001364. doi: 10.1371/journal.pgen.1001364 (PMC3077376; doi:10.1371/journal.pgen.1001364)
Supplement: Table S2 — Lipid metabolism-related genes and alleles used this study. (0.05 MB DOC) [file pgen.1001364.s007.doc]

**Supporting Table2.** Lipid metabolism-related genes and alleles used in this study

| ***Drosophila* gene** | **Human homolog** | **Allele** | **Strain number** | **Source** |
| --- | --- | --- | --- | --- |
| *CG9904/dSeipin* | Seipin | *RNAi* | V45478 | VDRC |
| *CG17608/fu12* | AGPAT1 | *RNAi* | V51079 | VDRC |
| *CG3812* | AGPAT2 | *RNAi* | V44418 | VDRC |
| *CG1049/cct1* |  | *Cct1^16919^* | 7319 | Bloomington |
| *CG8709* | Lipin | *RNAi* | V36007 | VDRC |
| *CG31991/mdy* | DGAT | *mdy^EY07280^(EP)* | 20167 | Bloomington |
| *CG31991/mdy* | DGAT | *mdy^qx25^* | 5095 | Bloomington |
| *CG5508* | GPAT | *CG5508^EY00734^ (EP)* | 20097 | Bloomington |
| *CG5508* | GPAT | *CG5508^e01407^* |  | Harvard |
| *CG9057/Lsd-2* |  | *Lsd-2^EY07971^ (EP)* | 19733 | Bloomington |
| *CG9057/Lsd-2* |  | *Lsd-2^KG00149^* | 13382 | Bloomington |
| *CG33131/SCAP* | SCAP | *SCAP^EY06708^ (EP)* | 16750 | Bloomington |
| *CG7962/CdsA* | CDS | *CdsA^1^* |  | Raghu et al., 2009 |
| *CG7962/CdsA* | CDS | *RNAi* | 7962-R1 | NIG-FLY |
| *CG7962/CdsA* | CDS | *CdsA^EY08412^ (EP)* | 16893 | Bloomington |
| *CG5295/bmm* | ATGL | *UAS-bmm* |  | Grönke et al., 2005 |
| *CG5295/bmm* | ATGL | *bmm ^1^* |  |

EP or UAS lines were used for gene overexpression. Other alleles were used for loss-of-function analysis.
