# Supplementary material for: Tissue-Autonomous Function of Drosophila Seipin in Preventing Ectopic Lipid Droplet Formation
Source: PLoS Genet. 2011 Apr 14;7(4):e1001364. doi: 10.1371/journal.pgen.1001364 (PMC3077376; doi:10.1371/journal.pgen.1001364)
Supplement: Table S3 — Primers for qRT-PCR. (0.04 MB DOC) [file pgen.1001364.s008.doc]

**Supporting table 3. Primers for qRT-PCR**

| Gene | Forward Primer | Reverse Primer |
| --- | --- | --- |
| *dSeipin* | ACGACCTGGTTGCCTACAG | CTGCGGGATTTCTTGACC |
| *GPAT* | CCTTTTGGCCTTCACTTCC | TCTCGATCTGCTTCGTGTTC |
| *AGPAT1* | CGCGAGACTATCGAAATGC | ACTGCCCCATGGTCCTTAC |
| *AGPAT2* | GCTGGCCTAATCTTCATCG | CCCACAGTTTGATACGTTGC |
| *Lipin* | GGGCATGAATGAAATCGAG | TCACCACCTTGTCGTTGTG |
| *DGAT/mdy* | CGTTCTCCAATATGGACGTG | AAAAGCAGAGCCAGCAAAG |
| *CdsA* | ATCATCGGGTTCGTTTGG | TGAGGTAACTCTGGGTGACG |
| *Cct1* | CACGCACCATCTCACAAAG | GACGTACTTCCGCTTGACG |
| *bmm* | TCCCTCCTTCAACATCCAG | TGTGCAGTCGTCCATTCAC |
| *Lsd-2* | GCAGTCTGGCTGTCAACG | CTCGCACTTGGGGAAGTAG |
